# Analytical Gas Sensing in the Terahertz Spectral Range

**DOI:** 10.3390/mi14111987

**Published:** 2023-10-27

**Authors:** Andreja Abina, Uroš Puc, Mojca Jazbinšek, Aleksander Zidanšek

**Affiliations:** 1 Jožef Stefan International Postgraduate School, Jamova cesta 39, SI-1000 Ljubljana, Slovenia; uros.puc@mps.si (U.P.); aleksander.zidansek@mps.si (A.Z.); 2 Institute of Computational Physics, Zurich University of Applied Sciences (ZHAW), Forschungsschwerpunkt Organic Electronics & Photovoltaics, Technikumstrasse 71, 8400 Winterthur, Switzerland; mojca.jazbinsek@zhaw.ch; 3 Department of Condensed Matter Physics, Jozef Stefan Institute, Jamova cesta 39, SI-1000 Ljubljana, Slovenia; 4 Department of Physics, Faculty of Natural Sciences and Mathematics, University of Maribor, Koroška cesta 160, SI-2000 Maribor, Slovenia

**Keywords:** terahertz spectroscopy, gas sensing, air pollution, environmental monitoring

## Abstract

Exploiting the terahertz (THz) part of the electromagnetic spectrum is attracting attention in various scientific and applied disciplines worldwide. THz technology has also revealed its potential as an effective tool for gas analysis in astronomy, biomedicine and chemical analysis. Recently, it has also become important in environmental applications for monitoring hazardous and toxic gases in the atmosphere. This paper gives an overview of THz gas detection analytical methods for environmental and biomedical applications, starting with a brief introduction to THz technology and an explanation of the interaction of THz radiation with gaseous species and the atmosphere. The review focuses on several gaseous species and groups of air pollutants that have been or can be analysed by THz spectrometry. The review concludes that different but complementary THz detection methods allow unique detection, identification and quantification of gaseous and particulate air pollutants with high selectivity, specificity and sensitivity. THz detection methods also allow further technological improvements and open new application possibilities.

## 1. Introduction

In recent decades, increased problems with air pollution have been observed in large areas and big cities in developed regions and developing countries around the globe. The effects of air pollution show consequences on the regional, continental and global scale. Additionally, the distribution of different pollutants by wind contributes to air pollutants reaching even rural and remote areas. This is especially important when an environmental accident or catastrophe occurs. Air pollutants have both natural and anthropogenic origins, but the major part is mainly contributed by industrial processes and motor vehicles. The airborne particles and the increased levels of toxic atmospheric gases seriously impact human health, other living organisms, ecosystems and the environment. In general, air pollution is: “A state of the atmosphere which leads to exposure of human beings and/or ecosystems to such high levels or loads of specific compounds or mixtures thereof that damage is caused” [1]. To perform rapid and targeted interventions, which would lead to a decrease in direct or indirect mortality due to air pollution, adequate sensing techniques must be developed and transferred into real applications. The growing awareness of air pollution is the driving force for new gas sensor development, which will play a key role in the future of air quality sensing. Commercial sensors for air pollution monitoring should be mobile, low cost, highly robust and enable greater spatial coverage for sensing and ease of production.

**Figure 1:**
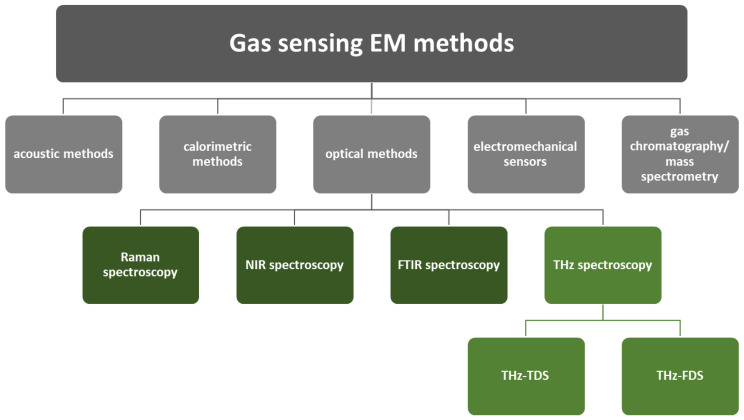
A diagram showing the division of EM gas-sensing methods into different methods, covering optical methods, which are further subdivided into spectroscopic methods, including THz spectroscopy. THz spectroscopy is also further divided into terahertz time-domain spectroscopy (THz-TDS) and terahertz frequency-domain spectroscopy (THz-FDS).

Accurate analysis of atmospheric gaseous species is critical for various applications, including environmental monitoring of Earth’s atmosphere for predictions of future climate changes and air quality [2], planetary missions [3], control of industrial chemical processes with gaseous intermediates, products or side-products [4], explosive vapour detection [5,6], breath analysis for medical applications [7] and detecting dangerous gases at disaster sites [8,9,10]. Gas-sensing methods for various applications have been systematically reviewed by Liu et al. [4]. Precise monitoring of gases in the atmosphere is crucial for researchers studying climate changes, atmospheric processes, ecology and other sciences. Reliable data allow a better understanding of complex systems and phenomena. Sensing methods can be divided into two major groups: the first is based on the variation of electrical properties and the second is based on other properties (Figure 1). The second group includes electromagnetic (EM) methods, including optical methods, acoustic methods, calorimetric methods, electromechanical sensors and gas chromatography, which is often combined with mass spectrometry (GC-MS). In this review, optical methods are of interest, especially those based on spectroscopy, which can be used for gas sensing due to its high sensitivity and characteristic fingerprints [11]. Optical methods can achieve higher sensitivity, selectivity and stability than non-optical methods, as well as enable online and real-time detection due to relatively short response time. Their applications as gas sensors for environmental monitoring are still limited mainly due to the complexity and size of the system as well as relatively high cost. The spectroscopic analysis of gases is often performed by using Raman spectroscopy [12,13], near-infrared (NIR) spectroscopy [14] or Fourier transform infrared spectroscopy (FTIR) [15]. Recently, novel spectroscopic techniques based on submillimetre (sub-mm) or terahertz (THz) radiation were used as an environmental monitoring system and gas detection and/or identification analytical tool.

THz spectroscopy has several advantages over other spectroscopic techniques. THz spectroscopy focuses on the low-frequency vibrational oscillations of molecules. This allows a more detailed investigation of low-frequency vibrations that are difficult to reach with other techniques. THz and FTIR spectroscopy are the closest approaches, where parts of the EM spectrum even overlap. THz spectroscopy uses the low-frequency range of THz waves, typically between 0.1 and 10 THz (or even higher up to 30 THz), which allows the analysis of vibrational and rotational vibrations of molecules in the low-frequency spectrum. FTIR spectroscopy, on the other hand, typically focuses on the range between 12 and 120 THz, i.e., on the analysis of vibrational oscillations of molecules in the mid-frequency spectrum. THz radiation penetrates relatively well through non-metallic materials such as plastics, rubber and some fabrics, while IR radiation has limited penetration through these materials, but penetrates well through thin layers. However, FTIR spectroscopy is more sophisticated in its development and practical use and is therefore more commonly applied in traditional laboratory applications. NIR spectroscopy uses light with an even higher frequency in the near infrared than FTIR, i.e., a frequency range between approximately 120 and 375 THz. NIR radiation therefore has even more limited penetration and is more suitable for surface measurements. NIR and FTIR spectroscopic systems are often less complex and more affordable, while THz spectroscopic equipment is often more delicate and requires special optical and electronic components and often longer measurement times. Raman spectroscopy is sensitive to changes in molecular vibrations and rotations, like THz spectroscopy, but is based on changes in the energy state of the molecules due to contact with laser light. The Raman dispersion phenomenon is excited by the laser light, and the scattered light is then analysed by a spectrometer. Raman spectroscopy requires surface sampling. However, all optical methods have their advantages and disadvantages, but each contributes unique results to gas analysis.

Terahertz technology has recently gained increased attention since it shows great potential in the detection and identification of chemical substances as well as in the imaging of suspicious objects and materials’ macrostructure characterisation [16,17,18,19]. The applications of the developed THz systems spread their roots in various scientific fields. Most research and application solutions are devoted to medicine and biomedicine [20,21,22], pharmaceutical product control [23], security [24], space exploration [3,25], polymer and biopolymer analysis [26,27,28], waste management [29,30] and communication technologies [31,32]. Some attempts were also made to study food and agricultural products [33], construction and building materials [34], art and archaeology [35] as well as environmental monitoring [36]. However, THz gas sensing is an emerging technology with several practical applications and unique advantages when compared to other mature gas-sensing technologies. It was used in security applications for the detection of explosives and hazardous materials, even identifying trace amounts of explosive compounds, and also in complex environments [37,38,39,40]. The sensitivity of THz radiation to several pollutants and greenhouse gases can be employed for the environmental monitoring of air quality [41,42,43]. The pharmaceutical and chemical industry can exploit the potential of THz gas sensing when controlling and ensuring the quality of pharmaceutical products or investigating the composition of gas mixtures [22,44]. THz radiation can penetrate biological tissues without ionising, therefore THz gas sensing can be applied as a non-invasive and label-free method for biological samples’ investigation in biology and biomedicine. The latter is very important for early disease detection and monitoring since the composition of exhaled breath can reveal the presence of specific volatile organic compounds (VOCs) associated with diseases, such as various cancers or diabetes [45,46,47]. Another practical application is in the food industry [48,49], where THz systems can detect gases released from food products to assess their freshness, ripeness and quality as well as faulty packaging. The changes in the gas composition emitted from meat, vegetables or fruits can provide early indicators of spoilage or microbial contamination. It is important to note that THz gas sensing is a unique option for a variety of potential applications, but the field is still evolving and there are still many challenges to overcome, such as the development of compact and affordable THz systems that can be used in real time and in real industrial environments.

THz spectroscopic techniques are just one branch of analytical gas-sensing methods, where EM radiation is transmitted through the gaseous sample usually caught in a cell or a chamber. Compared to other mature gas-sensing technologies, THz gas sensing offers several advantages. As mentioned before, THz radiation is non-ionising, which means it does not damage the biological samples or materials being analysed. THz methods cover a wide range of frequencies between microwaves and infrared radiation, allowing flexibility in the choice of frequency for a specific application and the analysis of different gases. THz sensors are highly sensitive with high spectral resolution and thus capable of identifying and detecting trace amounts of gases, even in hard environmental conditions such as following combustion processes or fire accidents, where the target gas has a high concentration (typically a few hundred ppm to a few tens in %) [39] but its analysis is still difficult because of mixing with the aerosol that disturbs the radiation path by light scattering. Bassi et al. demonstrated that this is not an obstacle for THz radiation which can penetrate through heavily sooted ethylene flame under elevated pressures up to 1.6 MPa [50]. THz spectroscopy offers several other advantages over other sensing techniques [34], among them the ability to gain vibrational information due to inter- and intramolecular modes and rotational information which is especially valuable for gaseous molecules’ characterisation. From the resulting spectrum, qualitative and quantitative spectroscopic analysis is possible. THz sensing can be contactless and non-destructive, which is advantageous for non-invasive analysis, where no gas sampling is required. This maintains the integrity of the samples and allows continuous monitoring of gas processes. One can rapidly and non-destructively recognise various gases and determine their concentrations even in multiple-component samples. Comparing THz methods with the other gas detection methods in Figure 1, it can be concluded that THz methods are better suited for accurate spectroscopy and real-time monitoring, while, e.g., acoustic methods offer ease of use and lower implementation costs. Most other methods, including calorimetric methods and mass spectrometry, involve sampling, which is often not necessary for THz analyses. The choice between THz and other methods depends on the specific requirements of the gas analysis. The methods are complementary as each offers unique advantages for different applications, but each also has its own disadvantages. In general, equipment for THz analysis of gases is comparable to or even more expensive than some other analytical techniques such as gas chromatography or mass spectrometry. However, it is important to consider not only the costs but also the performance of the equipment when comparing the methods. However, the price of THz systems has been decreasing since they first appeared on the market a few decades ago, making the technology more accessible for a variety of applications.

This article aims to review THz gas detection methods for environmental and biomedical applications. The review starts with a brief introduction of THz analytical methods and continues with an explanation of how THz radiation interacts with gases and the atmosphere. The review focuses on several groups of air pollutants and gaseous species (e.g., volatile compounds, nitrous oxide, alcohols, sulphur oxides, carbon oxides, nitriles, aldehydes, fine particulates, aromatic hydrocarbons and others) that have been or can be analysed by THz spectroscopy. The review concludes that THz spectroscopy has promising potential and offers several applications in environmental sensing, including gas detection and identification. In addition, the technology still opens several opportunities for further development and application.

## 2. Terahertz Analytical Methods

**Figure 2:**
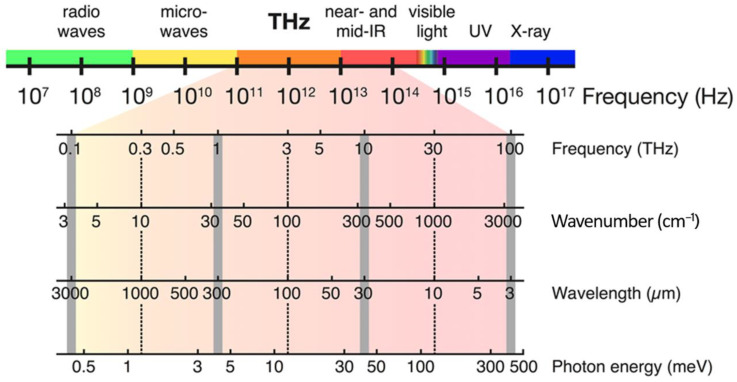
A schematic showing the extended terahertz (THz) band of the electromagnetic spectrum.

The THz band is not a precisely defined frequency range but is generally considered to span from approximately 0.1 THz (100 GHz) to 10 THz (10,000 GHz) in the EM spectrum, corresponding to wavelengths between 3 mm and 30 µm. As depicted in Figure 2, the position of the THz band within the electromagnetic spectrum is localised between the microwave (MW) and infrared (IR) regions. Some references indicate that the THz gap exists in the frequency range above MW frequencies (typically above 300 GHz) and below IR frequencies (typically below 30 THz). Therefore, at the edge frequencies, it is also possible to have an overlap of the THz gap with the MW band on one end and the IR band on the other end. In comparison to other analytical methods, THz spectroscopy is safe, non-destructive, contactless and reliable for real-time monitoring due to its lower photon energy and higher signal-to-noise ratio (SNR) [51]. The photon energy in this part of the electromagnetic spectrum corresponds with those of the vibrational, rotational and translational motions of many lightweight molecules and atoms [3]. 

In comparison to IR spectroscopy, THz radiation can probe not only intramolecular but also intermolecular vibrations in some molecules. Furthermore, IR spectral frequencies correspond only to vibrational modes, which are related to functional groups [52]. THz spectrometers are more sensitive below 3 THz [53] where the rotational modes of gases exist. THz waves can also penetrate through different media, e.g., concrete walls, plastics, ceramics, paper, wood and fabrics. For instance, the detection of carbon monoxide (CO) and nitric oxide (NO) at fire sites can be achieved with THz sensors, whereas IR gas detection in such situations is usually blocked by concrete walls [54,55,56]. With sub-mm wave measurements, much higher spectral resolution (*λ*/Δ*λ* > 10^6^) can be achieved due to the smaller absolute Doppler line broadening at lower frequencies. Spectral resolution is defined as a measure telling us how nearby features in wavelength space are separated. It is calculated as a ratio between the measured wavelength *λ* and the minimum wavelength separation Δ*λ* of two resolved features. In the case of gases, the rotations of molecules produce a broadening of spectral lines. Moreover, THz waves have a longer wavelength than IR and visible light. Therefore, they are scattered less by dust and smoke particles in the air [8]. Furthermore, sub-THz measurements are not affected by aerosol because the wavelengths of THz waves are much longer than the aerosol particles. Besides qualitative analysis, quantitative analysis is also possible, which requires a certain absorption line intensity. Some molecules, e.g., nitriles, exhibit much stronger absorption line intensities in sub-THz frequencies, thus, detection at lower concentrations is possible [57]. The quantification is more accurate if multiple absorption lines are included in the calculations [58]. As mentioned above, THz spectroscopy can collect qualitative and quantitative data in almost real time due to a relatively short response time [53]. All these characteristics of THz radiation bring THz technology into analytical science. 

Microwave spectroscopy that uses frequencies between 300 MHz and 300 GHz is also widely used for many polar gas recognitions. At microwave frequencies, heavy gas molecules exhibit their rotational resonances, whereas light gas molecules have rotational resonances in the mid-infrared region. The latter is generally complicated with rotational–vibrational modes. In comparison to THz waves, microwave radiation can reveal only a few characteristic features because the rotational translation modes in this region are relatively low [53]. Most gas molecules, however, do not have many resonance transitions at microwave frequencies. Moreover, THz waves occupy frequencies that are an order of magnitude higher in comparison to microwave- and- millimetre-wave bands. Therefore, their energy can be easily focused onto a small spot, which is especially desirable for THz imaging [8]. THz frequencies between the microwaves and the IR regions are therefore ideally positioned for most widespread gases because there are enough resonance transitions in the THz frequency range, but not too many.

In general, both passive and active THz methods are available for gas sensing. Passive methods detect the electromagnetic waves emitted by gas molecules, whereas active methods first illuminate the sample with THz waves from a certain distance and then analyse the detected signal transmitted or reflected from the sample. In some cases, such as fire sites, the background of the dangerous gases is usually lost in high-temperature objects. Therefore, active methods are more sensitive [8]. The active THz analytical methods for gas analysis can be divided into two modes (Figure 1): terahertz time-domain spectroscopy (THz-TDS) and terahertz frequency-domain spectroscopy (THz-FDS). THz-TDS systems mainly use femtosecond (fs) lasers, whereas other lasers and sources are usually included in CW-THz and THz-FDS systems [54]. THz-FDS is closely related to THz-TDS and measures the frequency-dependent complex dielectric properties of materials. The next subsections present a short description of both approaches, including a discussion about their pros and cons, which are summarised in Table 1. Considering the advantages of both techniques, one can conclude that THz-TDS and THz-FDS are complementary techniques that provide different types of information in the THz spectral region. THz-TDS provides broadband spectral information with high time resolution, while THz-FDS focuses on frequency-dependent dielectric properties.

### 2.1. THz-TDS

The time-domain signal carries different information, including phase information related to the dielectric properties of the sample and spectroscopic information that reflects the chemical composition of the sample [52]. Furthermore, with Fourier processing, the corresponding frequency-domain spectrum with unique spectral signatures is created, which is used for supplementary analysis [52]. Although THz-TDS has a wide spectral coverage even from 0.1 to 20 THz [59,60], it suffers from limited frequency resolution in the order of 1 GHz [61], which is frequently insufficient for distinguishing adjacent peaks that lie close together. An additional experimental problem is related to the instability of the entire system and the reproducibility of the measurements at constant humidity and temperature. However, the best measure of the accuracy is still the reproducibility [62].

**Figure 3:**
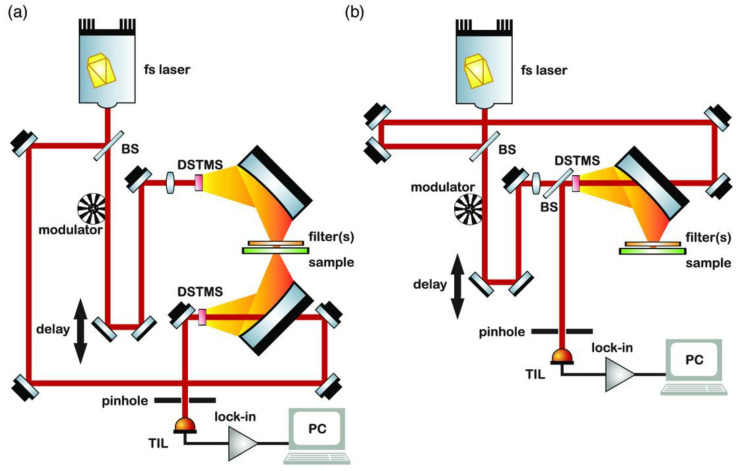
Schematic presentations of a typical THz-TDS system based on organic electro-optic (EO) crystal 4-*N*,*N*-dimethylamino-4′-*N*′-methyl-stilbazolium 2,4,6-trimethylbenzenesulfonate (DSTMS) in (**a**) transmission and (**b**) reflection geometry [59]. The incoming laser beam from a femtosecond (fs) laser is split at the beam splitter (BS) into the pump and the probe beam. The photodetector detects the signal proportional to the THz electric field by the THz-induced lensing (TIL) principle.

The typical principle of operation of a THz-TDS system transmission and reflection configuration is depicted in Figure 3. Here, ultrashort laser pulses (<100 fs) from a fs laser source are used and split into two laser beams at the beam splitter. The pump beam is used for THz generation, whereas the probe beam is used at the detector side as a gating pulse. In the case of the pump beam, the beam is focused on the THz generator, i.e., photoconductive antenna or electro-optic crystal where the THz pulse is generated. Either the pump beam or the probe beam is temporally delayed by using an optical delay line to ensure that the optical pulse at the THz detector arrives at the same time as the generated THz pulse and gates the detector. By changing the position of the optical delay line, the THz pulse is mapped out as a function of time. To further increase the detection sensitivity, an optical chopper modulates the pump beam and a lock-in amplifier extracts the THz-induced modulation on the probe beam [63]. The THz spectrum is obtained by Fourier transforming the acquired time-domain signal. Typically, a wideband spectrum of several THz can be obtained with a frequency resolution of 1 GHz or more [39]. Most THz-TDS systems are based on photoconductive antennas or electro-optic crystals. The basic principles of both are presented in the following paragraphs.

#### 2.1.1. Photoconductive Antennas

In THz-TDS systems, the most commonly used principle for generation and detection of THz pulses is based on photoconductive antennas. Photoconductive antennas generate and detect THz pulses by transient photocarriers induced with fs laser pulses. Typically, they consist of two metal electrodes deposited on a semiconductor substrate with a gap of a few µm between them. To emit the THz radiation, when a fs laser excites the gap between electrodes, a DC bias voltage must be applied between electrodes. Thus, the emission happens when the photon energy of the fs pulses is larger than the band gap of the semiconductor substrate to generate free electron and hole pairs in the gap between the electrodes. A DC bias field accelerates the free carriers and they produce photocurrent [63] and THz radiation. A similar principle of operation is applied in the detection regime. Instead of applying a DC bias voltage between electrodes, an ammeter is connected. The electric field on the photoconductive antenna can be measured by changing the time delay between the THz pulse and the laser probe pulse. The electric field of the photoconductive antenna can be mapped out at any given point in time by the probe pulse [63]. Modern designs of photoconductive antennas achieve a dynamic range of 90 dB, emitting power of a few µW and a typical wideband spectrum ranging from 0.05 THz to 2–6 THz [64].

#### 2.1.2. Electro-Optic Crystals

Optical rectification (OR) is a second-order non-linear optical effect that occurs in non-centrosymmetric non-linear crystals and is a difference frequency generation with a frequency difference close to zero [63]. Generation of THz radiation by ultrashort laser pulses (fs pulses) relies on efficient difference frequency mixing of all the frequency components within the spectrum of the laser pulse. As a result, a distribution of different frequencies in the time domain appears as an electric field transient with a shape like the envelope of the laser pulse. The most important factor in THz generation by OR is the matching between the group velocity of the fs laser pulse and the phase velocity of the THz field [63,65]. This phase/velocity matching defines the coherence length, which sets the optimal thickness of the non-linear crystal. When the non-linear crystal is used as a detector of the THz field, the electro-optical sampling process is commonly used. It is based on the Pockels effect where the polarisation of the optical probe pulse can be modulated by the THz pulse. The THz field provides a change in the refractive index along one axis of the electro-optic crystal, making it slightly birefringent. Therefore, its polarisation becomes slightly elliptical depending on the THz strength [66]. This polarisation change is converted to intensity change by an analyser, for example, a Wollaston prism. Usually, a pair of balanced photodiodes is used to map out the signal and suppress the noise. The THz bandwidth in optical rectification and electro-optic sampling is limited by two main factors—the pulse duration of the excitation laser and the phase-matching conditions [63]. With modern ultrafast lasers with pulses of a few fs and organic (4-(4-(N,N-dimethylamino)styryl)-1-methylpyridinium 4-methylbenzenesulfonate (DAST), 4-(4-(N,N-dimethylamino)styryl)-1-methylpyridinium 2,4,6-trimethylbenzenesulfonate (DSTMS), 2-(3-(4-hydroxystyryl)-5,5-dimethylcyclohex-2-enylidene)malononitrile (OH1)) as well as inorganic (zinc telluride (ZnTe), gallium selenide (GaSe), gallium phosphide (GaP)) electro-optic crystals, bandwidths of tens of THz to over 100 THz were achieved [59,67,68,69].

### 2.2. THz-FDS

**Figure 4:**
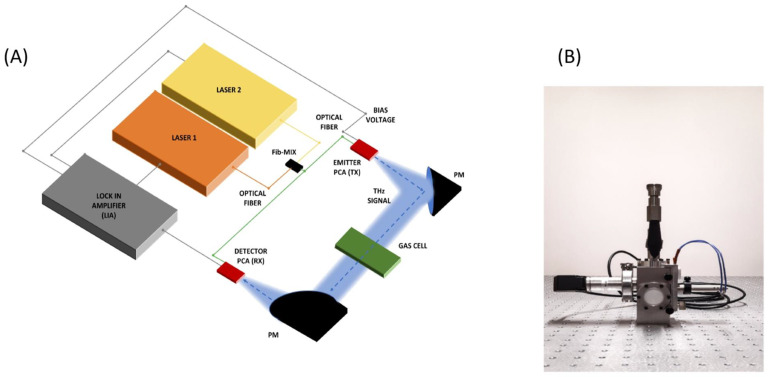
Schematic layout of the THz-FDS system for gas detection (**A**) with a gas absorption cell (**B**) [41]. The THz system includes two distributed feedback (DFB) lasers, one THz transmitter (TX) and THz receiver (RX), four off-axis parabolic mirrors (PMs), laser combiner (Fib-MIX) and a signal-processing unit. THz radiation is generated by a photoconductive antenna (PCA). © 2022 Optica Publishing Group under the terms of the Optica Open Access Publishing Agreement (https://doi.org/10.1364/OE.456022), accessed on 3 September 2023.

THz-TDS is most commonly used for the analysis of various materials. In the case of gases, however, the shortcomings of such systems are evident, as the achievable spectral resolution is limited. THz-FDS is closely related to THz-TDS in physical processes, measurement and detection schemes (Figure 4). The main advantages over THz-TDS are the high-frequency resolution (~MHz), the possibility to work at a fixed frequency or in a tunable frequency range and the relatively low cost. THz-FDS does not require mechanical movements. Therefore, such systems are also more stable, compact and portable. All this allows them to perform real-time measurements in situ [41]. In THz-FDS, a spectrum is obtained by frequency-scanning narrowband CW-THz radiation [39]. 

In THz-FDS or THz-TDS, the source of THz radiation over a wide frequency range can be a femtosecond laser or a more specialised THz emitter such as a quantum cascade laser (QCL), free electron lasers (FELs) or a backward wave oscillator (BWO). QCLs are well suited for high-resolution molecular spectroscopy in the THz range, but their use is still limited by the size and complexity of the system, which requires cooling. Hagelschuer et al. have proposed a real-time gas detection system with QCLs, without the need for liquid-helium-cooled detectors, allowing fast measurements with a time of 10 ms per spectrum and real-time gas concentration measurements at a frequency of 100 Hz [70]. The method is based on modulation of the length of the external cavity and exploits the intermediate optical feedback regime which causes a change in the frequency of the QCL and its terminal voltage. The THz-FDS technique with BWOs is also called fast submillimetre scanning spectroscopy (FASSST), which can cover a wider THz frequency range, but not all of it. BWO systems are not always ideal for the analysis of gas molecules at atmospheric pressure due to the mismatch between the narrow CW-THz linewidth and the atmospherically broadened absorption lines. Its use is more promising for high-resolution spectroscopy of gas molecules at low pressure [39]. For precise tuning of the THz frequency, optical parametric oscillators (OPOs) can be applied. In recent years, free electron lasers (FELs) have become a valuable source to generate high-power coherent THz radiation. FELs are typically synchrotron-based devices that use high-energy electron beams. Their tunability is achieved by adjusting the strength of the magnetic field in the undulator, which forces the electrons into a periodic motion, resulting in the generation of synchrotron radiation at the desired wavelength. Less common but also possible is the use of gas lasers, e.g., CO_2_ lasers, as a THz source. To summarise, QCLs are more portable and compact sensors, allowing for precise control of the emitted THz frequencies, and offer higher power and broad spectral range but have limited output power at some THz frequencies. BWOs provide high output power but are less portable and require cryogenic cooling. Thus, they are more suitable for laboratory analysis. In comparison to QCLs, they have limited tunability. FELs offer the highest power and tunability through the entire THz frequency spectrum, but their use is usually limited to well-equipped synchrotron research centres due to high equipment costs and size. BWOs typically operate in a limited frequency range and are therefore less selective and specific in gas analysis compared to FELs or QCLs, but they can still provide selectivity if the target gas has resonances in their operating frequency range. In general, FELs can provide high sensitivity, selectivity and specificity due to their tunability over a wide THz frequency range to match specific molecular transitions of gases. The most suitable source for gas detection is difficult to determine, as the choice depends not only on the purpose of the survey and the target gas properties but also on several other factors such as the required spectral range, power, portability and budget constraints.

Unlike THz-TDS, where time-domain data are acquired, THz-FDS modulates the frequency of the THz radiation. Sinusoidal modulation is typically applied to the THz source, resulting in frequency-modulated THz radiation. In THz-FDS, a bolometer or Golay cell is used as a THz detector, measuring the amplitude and phase of the transmitted or reflected THz signal at different modulation frequencies. The detector response is thus recorded as a function of the modulation frequency. From the data obtained, the complex dielectric properties of the sample are calculated, including its refractive index and absorption coefficient as functions of frequency. 

The principle used to generate CW-THz radiation can be based on optical technology, such as photomixing, difference frequency generation (DFG) and FIR gas lasers, all-solid-state technology, like diode-based multipliers, p-type germanium lasers and QCLs or based on free-electron sources, including backward wave oscillators and free-electron lasers [71]. As reported by Martin-Drumel et al., the continuous-wave terahertz (CW-THz) photomixing technique has the greatest tuning frequency range of any known coherent source in the THz region. It thus ensures a spectral purity lower than the Doppler limit at room temperature [54]. Photomixing or optical heterodyning is a down-conversion technique where two tunable, dual-frequency, CW laser sources are used to excite a photoconductive antenna (i.e., LT-GaAs) to generate CW-THz radiation. The tuning range can be up to a few THz with a high-frequency resolution reaching sub-MHz [58]. However, the output power is relatively low, in the range of a few µW [71]. In the case of DFG, which is a second-order optical process occurring in non-centrosymmetric crystals, two narrowband laser beams with slightly different frequencies like in photomixing are used. When the two optical beams co-propagate and are linearly polarised in the same direction, their interference generates a beat, which oscillates with the difference frequency between the beams. The second-order non-linear polarisation of DFG is proportional to the beat intensity and, consequently, the THz radiation field is induced by the non-linear polarisation. With DFG, frequency tunability up to several tens of THz was demonstrated [71]. Another type of optical device is the FIR gas laser, where molecular gases are used as gain media (such as fluoromethane, difluoromethane, methanol and ammonia) for THz generation, which originates from the rotational transitions of the molecules. They produce discrete THz lines and, depending on the gain medium, FIR gas lasers are capable of producing a few mW of output at THz frequencies [71]. 

All-solid-state technologies, like the p-type germanium laser (p-Ge), a tunable laser operating in crossed electric and magnetic fields at liquid helium temperatures, are also promising. Continuous tunability in p-Ge lasers over the spectral range from 1–4 THz can be achieved by changing the applied electric/magnetic fields or through the introduction of intracavity elements. A typical average THz output power can be up to a few watts [71]. Diode-based frequency-multiplied microwave sources provide fast sweeping and high spectral purity without mode hopping [39]. They use the output from a microwave synthesiser at around 100 GHz and multiply it by using Schottky barrier diodes. The output power depends on the output frequency; usually, it is in mW at hundreds of GHz and in µW above the THz range [71]. The tunability is limited between 10% and 20% of the carrier frequency [39]. The QCL is a semiconductor laser. Laser action arises from transitions between electronic subbands formed in a series of quantum wells and, by “cascading” a number of such active regions together, the injected electrons undergo multiple lasing transitions as they pass through the device [64]. One of the limitations of QCLs is that they need cryogenic temperatures for operation, although there were several reports of emission in the 160–190 K range [71]. Modern THz QCLs have limited tunability and cover frequency lines in the range of 1–5 THz. Emitting power greater than 1 W was demonstrated [64]. BWOs are electron vacuum devices in which an electron beam interacts with a travelling electromagnetic wave. By using a slow-wave structure, the electrons are slowed down and the kinetic energy of the electrons is transferred to the electromagnetic wave. They operate in the range between 0.03 and 1 THz; however, several tubes have to be used to cover the whole range, as the tunability is about 10% around the central frequency. The power at frequencies below 100 GHz is more than 100 mW and at 1 THz around 1 mW [71]. In the case of FELs, where coherent radiation is produced from a beam of free electrons optically amplified in an undulator, the emitted wavelengths can vary from THz to X-rays. FELs operate in pulsed mode and can achieve THz power reaching megawatt levels with frequencies ranging from hundreds of GHz to 100 THz [71,72].

Over the past few years, metamaterials, which are artificial materials designed to manipulate electromagnetic waves, have been used in the field of THz research and system development. They can be customised to resonate at specific THz frequencies, allowing THz radiation to be limited. Examples of structures based on metamaterials are split-ring resonators and photonic crystals used to create THz cavities, which enhance THz radiation and allow higher sensitivity in gas detection. In recent years, research into plasmons [73] and cavities [74,75,76] has made an important contribution to the analysis of THz gases by confining and amplifying the THz radiation and increasing the sensitivity and selectivity of the measurements. Plasmons are collective oscillations of electrons on a noble metal surface. In THz gas sensing, they are used to enhance the interaction between THz radiation and gas molecules. When the frequency of the incident THz radiation matches the resonant frequency of the surface plasmons, a strong electric field is generated on the metal surface. This enhanced field acts on gas molecules near the surface, causing changes in THz transmission or reflection that can be detected and analysed. Another principle for improving gas analysis is cavities. These are structures designed to confine THz radiation in a small volume and to increase the interaction length between THz radiation and the investigated gaseous sample. When gas molecules are trapped in the microcavity, their presence modifies the THz field in the cavity. This change in field intensity or resonant frequency can be used to detect and quantify gas concentration. Some QCLs are designed with built-in cavities that increase the output power and spectral purity of THz radiation. Researchers often explore dual-mode resonators using both plasmonics and cavity structures to optimise their THz devices. This approach thus combines the advantages of plasmon-based and cavity-based detection, allowing for improved sensitivity and selectivity in gas analysis. By incorporating plasmonic structures, cavities and metamaterials into THz gas sensors, higher sensitivity, lower detection limits and better distinctions between different types of gases can be achieved, resulting in improved practical applications in areas such as environmental monitoring, safety and product quality control in industrial processes. 

Other approaches and technologies are being developed to improve gas detection using THz methods. Nanostructures can be used to increase the surface area that enhances the interactions between THz radiation and gases, acting as a substrate for attaching gas molecules. Of the nanostructures, much research is being carried out on graphene, which can be shaped to selectively detect certain gases. In one experiment, a subnanometre-thick monolayer of graphene showed strong metallic and plasmonic behaviour in the THz frequency range. This plasmon effect was significantly modified when the graphene layer was placed under a magnetic field of appropriate strength, which can be exploited to achieve higher-sensitivity gas detection [77]. Microfluidics is used for more precise manipulation of the gases [78]. By integrating different components of a THz system on a single chip, a compact and portable system could be possible for use in field applications [79]. In addition, the use of artificial intelligence and integration with chemometrics will be an improvement, allowing better understanding and analysis of THz spectral data, leading to improved identification and qualitative and quantitative evaluation of gases [22,80,81,82].

### 2.3. Gas Cells and Gas Preconcentrator

In general, a THz spectrometer consists of a THz radiation source, a detector and optical elements. Because the hydrostatic pressure can influence the sample’s properties, a gas absorption cell has been introduced in many spectroscopic experiments for gases as shown in Figure 4 [41,83,84,85]. Thus, during the measurements, the absorption cell is filled with the gaseous sample at a particular pressure. The emitted THz beam propagates through the cell filled with gas which absorbs certain frequencies [86]. The absorption and dispersion response of the gas sample is obtained by measuring the THz waveform for both an evacuated absorption cell and a cell filled with a sample gas at a specified pressure by using Fourier analysis techniques [87]. Recently, the advantages of measuring pressure-dependent spectra in the THz range have been recognised by numerous researchers [85,88]. 

A THz gas measurement system includes a well-designed gas cell with adequate propagation length and wall thickness. Longer paths can be achieved with folded absorption cells [47]. The folded absorption cell contains deflection mirrors to achieve an appropriate beam path length, usually above 1 m [9,86]. The long cells are needed to increase the interaction length between the THz radiation and gas molecules [74]. One experiment showed that the reduction of absorption cell length from 1.9 m to 0.56 m reduces the sensitivity of the THz sensor by a factor of 7.5 [7]. The material for gas cell design is also carefully selected because some measurements demand sample heating while others cooling. Usually, crystal quartz [89] or high-specific-resistance [61] silicon is selected as a material for the gas cell. The gas pressure within the cell must be precisely controlled, for instance, with a turbo-molecular pump, valves and a pressure gauge [7,86]. The obtained THz signals are sensitive to variations in atmospheric conditions, thus the gas cell and system optics chamber are evacuated individually to provide stable and reproducible measurements throughout longer periods [9,90]. In some cases, the cell must ensure evacuation down to 100 Pa to reduce the pressure broadening of the absorption lines for a given gas species [9]. 

Moreover, the THz chemical sensors operating at pressures below 1 Pa ensure that substances, which strongly interact with THz radiation, can be identified in the total amount of sample well below the picomol level, which is competitive with GC-MS, where sensitivity is around 100 femtomols. Besides this comparable achievement, the THz sensor has another advantageous specificity: it can operate without a cryogenic system and requires the processing of a much smaller amount of samples [47]. Thus, real-time applications are much closer to realisation.

An investigated gas is usually diluted in air under atmospheric conditions. Therefore, the gas must be first concentrated by a gas preconcentrator that acts as an absorbent for gas molecules. Simultaneously, other air constituents such as nitrogen (N_2_), oxygen (O_2_), carbon dioxide (CO_2_), argon (Ar) and water (H_2_O) are filtered out. For instance, a preconcentrator reduced the water concentration in the absorption cell by a factor of several hundred [47]. Sometimes additional thermal desorption of gases is necessary, which demands heating the preconcentrator [86]. The gas concentration system based on absorption and thermal desorption, e.g., porous carbon nanosieves [22], allows the identification of small substance amounts. Thus, by using a preconcentrator system, the sensitivity can be improved by up to five orders of magnitude [7,84]. Other preconcentrator techniques were also tested, including membranes, ion traps, plot columns and three-dimensional (3D) as well as two-dimensional (2D) adsorbents [84]. The latter showed the best performance, since less power is needed for heating, the thermal mass is reduced and the operational speed is higher [84]. Another noteworthy characteristic of the absorber is that it should not trap water because water vapour inside the absorption cell increases the dilution of trace gases. Zhang and Grischkowsky used THz-TDS to demonstrate the strong adsorption effect of silica aerogel for the vapours of water, heavy water, ammonia, methyl chloride and methyl fluoride [91]. In this study, the hydrophilic Si-OH groups of aerogels, which are more likely to bond with strongly polarised molecules, were passivated or replaced by the absorbed molecules of vapour compounds, which resulted in a decreased absorption in the frequency range above 1 THz. The other two gas samples tested, H_2_ and CO, showed no noticeable change in THz absorption spectra. However, the choice of absorbent depends on sample species selection in a given application. 

## 3. THz Detection of Air Pollutants

### 3.1. Interaction of THz Radiation with Gas Matter

Since one of the major applications of THz spectroscopy is material characterisation, it is important to understand the interactions between the employed electromagnetic radiation and the material under investigation. Understanding this aspect is necessary for the interpretation of obtained THz spectra. In the THz region, many absorption lines of gas molecules appear due to molecular rotational transitions and internal rotations as well as torsional vibrations [52]. Furthermore, the THz spectra of gases exhibit narrow lines with few distortions and errors. The linewidths are far narrower than the spectral resolution of the measurements [83]. The rotational spectra are redundant because they comprise hundreds or thousands of roughly periodic spectral lines [84]. This fact is advantageous for gas spectroscopy since a THz sensor does not need to cover the entire THz region. The density of spectral lines varies from one gas species to another. Most often, a 30 GHz wide spectral range contains enough spectral lines for the characterisation of any molecule with at least three atoms heavier than boron [84]. Thus, “white space” without lines can be neglected and the speed of THz measurements can be significantly improved.

The molecule must have a permanent dipole moment if someone wants to measure the rotational absorption resonances in the THz range [89]. Among all the predominant atmospheric molecules, water and water’s isotopes have permanent dipole moments and are therefore known to exhibit prominent THz rotational spectra. Molecules such as CO_2_ and O_2_ are also diatomic molecules with linear symmetry but they have a permanent dipole moment because of the unequal sharing of electrons between atoms, leading to partial positive and negative charges. This dipole moment is essential for rotational transitions. Some molecules, such as diatomic nitrogen, have linear symmetry with a symmetrical distribution of electrons, resulting in a symmetric charge distribution, which prevents them from forming a permanent dipole moment. Therefore, nitrogen does not exhibit rotational absorption in the THz range, and the THz rotational absorption for O_2_ and CO_2_ is also not significant. Carbon monoxide (CO) is also a linear diatomic molecule but it has a permanent dipole moment for the same reason as O_2_ and CO_2_. Moreover, the greater the dipole moment, the more significant the changes in energy levels, and the transitions between these levels produce absorption lines in the THz range. Thus, the rotational transitions in molecules like CO are quantised due to the energy level structure. The energy difference between adjacent rotational levels corresponds to THz frequencies and leads to spectral lines in the THz spectrum. Molecules with weaker dipole moments like CO may exhibit limited absorption in the THz frequency range [89] due to the limited resolution of the THz measurement system [61]. The absorption behaviour of molecules in the THz range is influenced by factors such as molecular structure and energy levels.

Each molecular species including gases absorbs THz waves in a unique spectral pattern showing not only the absorption features of the investigated molecule but often also the effect of the surrounding environment. However, absorption features are generally unique to each molecular species. For gases, pure rotational transitions are observed as Lorentzian resonances at distinct frequencies in the THz spectrum; therefore, the spectrum for each gas is unique [53].

THz waves can strongly interact with the rotational transitions that exist in a gas phase of substances. To obtain the response of this interaction, the measurements of gas samples require a long interaction length to obtain the appropriate absorption and high resolution. For this reason, the gas is measured in the absorption cell with a transparent window, which also neglects the absorption lines of water vapour. Usually, the polar water molecules absorb a fraction of the THz intensity, which is expressed as a reduced peak amplitude in the time-domain waveform and as a cut out of the particular frequencies in a frequency domain [92]. The water absorption lines can be removed numerically after measurements. The better way to avoid this influence is to perform measurements after evacuation in a dry air or nitrogen atmosphere. Additionally, the THz measurements also require information about the cell length and pressure due to the pressure-dependent spectral linewidths [92], which are also dependent on the system resolution. The spectral intensities scale linearly with the pressure [47].

From the measured THz data, the absorption coefficient and the dispersive phase can be independently derived from the time-domain waveform by a Fourier transform (FT). A rapid gas identification can be performed even without the FT because the rotational constants can be determined from the periodic recurrences in the time-domain waveform. Besides the identification, the quantitative analysis of gases is possible. When knowing the absorption coefficient and the absorption length within the cell, it is easy to determine the concentration of gas in the ppm range [92]. Thus, THz-TDS allows both single-species identification and quantitative analysis of gas mixtures.

### 3.2. THz Radiation Interaction with Atmosphere

Besides infrared and microwave sensors, several THz sensors have been included in orbital instruments for applications in astrophysics, cosmology as well as pollution and climate monitoring in the Earth’s atmosphere [3,57]. In particular, THz measurements improve understanding of dynamic processes in the Earth’s atmosphere, such as pollutant distribution, radiation balance, temperature profiling, global warming and ozone depletion [93]. For instance, a heterodyne radiometer on NASA’s Aura satellite, which was launched in July 2004, measured the thermal emission at sub-THz frequencies (118, 190, 240 and 640 GHz) as well as at 2.5 THz. By this space mission, the THz heterodyne radiometer/spectrometer system has been proven as a useful technique to measure trace constituent abundances and physical properties under various climate conditions. The THz technique allows the detection of the trace species with polar molecules at THz frequencies at very high sensitivities, i.e., at parts per trillion to parts per billion [57]. Additionally, THz instruments for planetary missions investigate the sources of trace gases and the concentration of key constituents in the planetary atmospheres in various weather conditions [57,94]. All these approaches and acquired data can be undertaken for environmental applications to better understand global warming, meteorological forecasting and atmospheric composition that affects climate and to study the pollution in the upper troposphere. 

**Figure 5:**
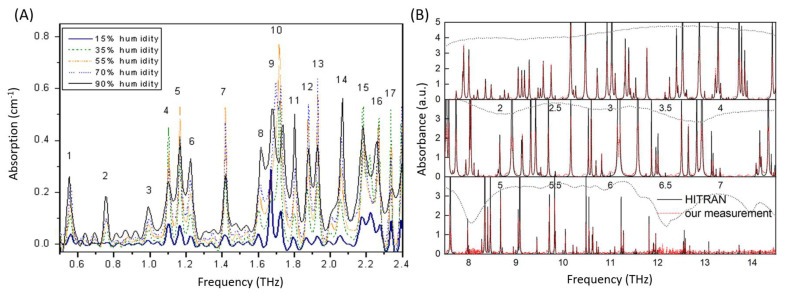
(**A**) THz absorption spectra of the atmosphere at different relative humidities at low THz frequencies [100] and (**B**) water vapour absorption lines (absorbance in arbitrary units, a.u.) with the high resolution of 2.7 GHz in comparison to the results from the HITRAN database in the frequency interval 1.5–14.5 THz [59] ((**A**) is reprinted from “Terahertz absorption spectrum of para and ortho water vapours at different humidities at room temperature,” *Journal of Applied Physics*, vol. 100, p. 094905, 2006, with the permission of AIP Publishing via Copyright Clearance Center Inc., https://www.copyright.com/, (accessed on 27 September 2023)).

When THz radiation propagates through the atmosphere, its amplitude and phase change due to several reasons. It is essential to understand all atmospheric effects [95], but herein we emphasise some of them with crucial importance: humidity, altitude, pressure, gas components and temperature. The polar molecules like water vapour are great absorbers of THz radiation and lead to ubiquitous absorption lines through most of the THz range (Figure 5B). Therefore, the THz atmospheric absorption is principally dominated by water vapour, which controls the electromagnetic propagation of broadband and narrowband THz waves in the atmosphere [62,96]. An increase in water content within the atmosphere decreases the transmission of THz radiation and results in narrower and shallower THz transmission windows as presented in Figure 5A [89]. Some researchers reported two promising areas between 1 and 2 THz that show a window of very little water absorption. The first area is located between 1.2 and 1.4 THz, the second one was identified between 1.4 and 1.6 THz [89]. Other authors reported an important transmission window below 1 THz in the range from 0.2 to 0.3 THz [96]. This results in a long atmospheric THz propagation path [89]. For this reason, the propagation path of the THz beam is often placed within a chamber with a nitrogen-purged environment to prevent the effects of atmospheric water vapour on the THz spectrum [89]. By comparing the THz spectrum of pure nitrogen and the THz spectrum of the atmosphere with usual relative humidity, one can notice the reduction in the peak amplitude of the main pulse and the modulation of the THz waveform due to the absorption and dispersion of THz radiation on water vapour molecules [89]. Moreover, water vapour absorption is usually described as a two-phase phenomenon. The first phase is the consequence of the absorption due to the resonant rotational or vibrational lines and the second is due to the continuum. Continuum absorption is most often defined as the difference between the experimentally observed spectrum and the calculated resonant absorption spectrum [97]. Knowing the location and width of windows with high atmospheric THz transmission is important for additional system developments as well as novel applications. For environmental monitoring, one must also consider the fact that the humidity changes with the weather conditions, changing seasons, altitude and geographical location [98].

Besides relative humidity, the propagation of THz radiation through the atmosphere also changes at different altitudes. At higher altitudes, the propagation of THz waves improves dramatically mainly due to the decrease in the humidity and temperature changes [89]. Some theoretical calculations demonstrated that the transmission window increases from 25% to 99% in the frequency range between 0.3 to 12 THz if altitude is changed from 4.2 km to 41 km [89]. A special THz-CW system has been developed that allows the detection of water vapour in the atmosphere at concentrations in the ppm range without the need for an absorption gas cell. Such a system uses a THz disk microresonator which can be further optimised to achieve even higher frequency resolution and sensitivity [99]. The proposed solution suggests the possibility of a compact and highly sensitive THz spectrometer for gas phase analysis, which would contribute to a wide range of applications.

The next atmospheric parameter, which has a major impact on THz measurements, is the atmospheric pressure. The pressure broadening of the lines limits the experimental resolution [89]. The spectral resolution of the selected THz system must be in the order of or less than the pressure broadening for the selected investigated gas molecules [22]. At pressures below 10 Pa, absorption linewidths of 4 to 5 MHz can be achieved with a heterodyne detection [101]. Spectral resolution of about kHz is necessary to obtain high selectivity of THz analysis in gas detection, quantification and monitoring in gas mixtures. To obtain this spectral resolution, two other conditions must be satisfied, i.e., Doppler line resolution (~10^−6^) and frequency measurements with accuracy of 10^−8^–10^−10^ [90]. At room temperature, the Doppler linewidth resolution is about 1 MHz at THz frequencies, therefore pressure should be kept from 0.13–6.67 Pa to achieve the best possible sensitivity and specificity of chemical spectroscopic analysis [47]. In addition, high selectivity can be achieved with a low-pressure gas cell and a high-resolution spectrometer [9]. For high-resolution measurements, it is preferable that the THz source is narrowband, i.e., the emission linewidth is much smaller than the linewidth of the transition [7]. The gas pressure importantly affects the refractive index, which increases with increasing pressure [88]. At lower pressures, the most relevant are techniques photomixing, frequency multiplication or FIR laser side-band spectroscopy [102,103].

THz radiation is attenuated not only by absorption but also by scattering of aerosols along the optical path. Aerosol particles in the atmosphere are dust, salts, ice particles, smoke particles, fumes and water droplets [39]. The value of a complex refractive index depends on the particle size distribution and the wavelength of incident radiation. When performing measurements through the atmosphere, one must consider both the Rayleigh scattering and the Mie scattering. Since the wavelength of THz waves is in the order of the size of aerosols, only Mie scattering should be taken into consideration [95]. Therefore, with THz spectroscopy one can perform direct measurements of samples contaminated with aerosols since the minimum wavelength in the THz range (e.g., λ ≥ 100 µm below 3 THz) is at least three orders of magnitude greater than the size of aerosol particles, whereas IR spectroscopy requires prefiltration to remove particles from aerosols [61].

Understanding THz propagation through the atmosphere is crucial for further development of sensing analytical tools in environmental applications. The knowledge about atmospheric attenuation of THz radiation is also important for selecting the optimal frequency bands for sensing systems. In addition, a gas THz spectroscopic database is necessary to discriminate between the atmospheric components. There are already several spectroscopic databases for gases such as HITRAN, Jet Propulsion Laboratory (JPL), Cologne Database for Molecular Spectroscopy (CDMS), SOA Terahertz Toolbox and Gestion et Etude des Informations Spectroscopiques Atmospheriques (GEISA) [53,89,95], which should be further upgraded with new or extended THz spectra.

### 3.3. THz Detection of Gases and Various Air Pollutants 

**Figure 6:**
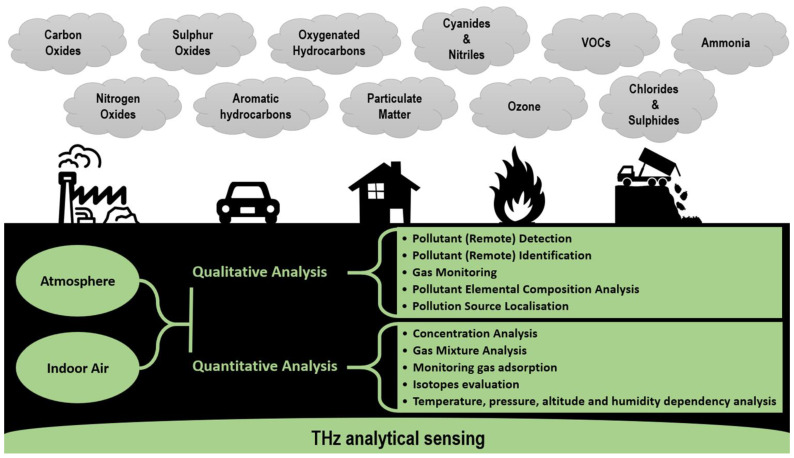
THz gas sensing for qualitative and quantitative analysis in environmental applications.

Many types of research have already proved that spectroscopy in the THz region is a powerful tool for the chemical analysis of various gaseous substances (Figure 6). Gas molecules can be measured by THz systems directly under atmospheric conditions or indirectly through isolated systems or through respiratory gases [22]. The atmospheric pollutants with small molecules are ideal candidates for THz spectroscopy because they exhibit strong transitions in this frequency range. Among them, the most interesting are hydrogen sulphide (H_2_S), carbonyl sulphide (OCS), formaldehyde (H_2_CO) and ammonia (NH_3_), which are important gases in various industrial, environmental and biological processes. For the gaseous species with larger molecules such as methanol (CH_3_OH), toluene (C_7_H_8_) and acetone ((CH_3_)_2_CO), internal rotations of the molecules produce many overlapping spectral peaks, which are hard to resolve by existing spectrometers. Other macromolecules like polycyclic aromatic hydrocarbons (PAHs) require spectrometers with a high sensitivity [58]. Simple molecules with diatomic structure and short molecular chains such as CO, H_2_CO and OCS have only a few absorption lines in a relatively narrow spectral band in the THz range, whereas larger molecules have up to 600 or even more transitions in the same frequency range [7]. 

**Figure 7:**
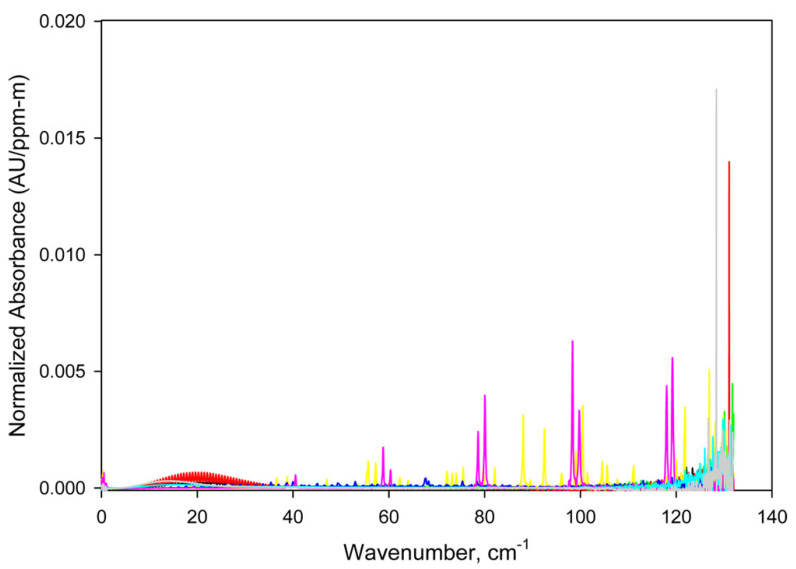
THz spectra of selected gases: acetaldehyde (black), acetonitrile (red), ethanol (green), water (yellow), methanol (blue), ammonia (magenta), propionaldehyde (cyan) and propionitrile (grey) in the frequency range from 0–140 cm^−1^, corresponding to the frequency range 0–4 THz. (Reprinted with permission from [52]. Copyright 2015 American Chemical Society).

Some gases exhibit equidistantly spaced absorption peaks, whose periodicity is different for various gases depending on the molecular structure and corresponding rotational modes [104]. The periodicity is related to the momentum of inertia of gas molecules. Thus, knowing the structure of gas molecules one can predict the interval between the equidistant absorption peaks. Due to unique spectral fingerprints, THz spectroscopy can discriminate between various gases (Figure 7). For instance, Hindle et al. demonstrated that a THz system is capable of discriminating between multiple species in cigarette smoke [58]. They recognised spectral signatures of hydrogen cyanide (HCN), CO and H_2_O at 2000 Pa and H_2_CO below 200 Pa. On the other hand, THz spectroscopy can be applied for the detection and identification of hazardous and toxic substances that may be formed at accidental sites or remain in construction materials after disasters [105]. Several toxic gases, e.g., HCN, CO, H_2_S, NH_3_, hydrogen chloride (HCl), acetonitrile (CH_3_CN), and nitrous oxide (N_2_O), exhibit rotational spectra in the THz band. 

This review focuses on experiments carried out to study the spectra of various gases and air constituents that can be considered convenient for environmental and biomedical applications. References included in the review are summarised in Table 2, which also provides a brief description of experiments considering the gas samples under investigation, the selected THz system and operating frequency range as well as the purpose of the study. For each gas, the Introduction gives a brief overview of why these gases are important in different applications. For selected gases, the lower detection limit is also given in Section 4. The review in the following subsections summarises recent advances and potential applications of THz spectroscopy in gas analysis and identifies challenges and directions for further research.

#### 3.3.1. Alcohols, Aldehydes and Ketones

Alcohols, aldehydes and ketones are oxygenated hydrocarbons often recognised as ubiquitous air pollutants. Although experimental studies have shown that alcohol fuels produce fewer emissions than unleaded gasoline fuel, some studies revealed that alcohol-based blended fuels have a higher fuel consumption rate and CO_2_ emissions [106]. On the other hand, aldehydes and ketones are major components of indoor air pollution and therefore their monitoring in industrial applications is crucial. 

Schmaltz et al. measured 75 and 17 absorption lines for methanol in the frequency range 238–252 GHz and 491–498 GHz, respectively [7]. For acetone ((CH_3_)_2_CO), the number of absorption lines in the lower frequency range from 238–252 GHz is 21 and 62 in the frequency range 491–498 GHz. In the case of other alcohols with a longer chain and OH functional group at non-terminal sides, this number increases dramatically, e.g., 2-propanol ((CH_3_)_2_CHOH) exhibited 826 absorption lines in the frequency range 238–252 GHz. In the case of ethanol (CH_3_CH_2_OH), Zhu et al. [107] observed that hydrogen bonding causes molecular changes on a picosecond timescale, which can also be noticed as fluctuations in THz spectra recorded during some periods. Smith and Arnold calculated the selectivity coefficient for methanol and ethanol by comparing the THz and IR absorption spectra after the path length and concentration normalisation [52]. Due to the molecular nature of the transitions at THz frequencies, the selectivity is greater for THz spectroscopy in comparison to functional group transitions obtainable by IR spectroscopy. Additionally, methanol was included in an experiment in which Graber et al. showed that SNR increases with the gas sample concentration [108]. In this study, they also observed that the methanol molecule with an asymmetric top shows a noteworthy variation between experimental and simulated spectral line intensities, although their resonance frequencies agree very well. More recently, research has also focused on n-propanol gas, the analysis of which is important for environmental monitoring and respiratory breath analysis in lung cancer patients. Lin et al. have developed a rapid method for the detection of this gas based on molecularly imprinted polymers (MIPs) and THz metasurface sensors. Since MIP adsorbed with n-propanol changes the dielectric environment of the sensor, the resonant frequency of the sensor also changes, which can be used to determine n-propanol concentrations in the range of 50 to 500 ppm [109].

Aldehydes are found as environmental pollutants with especially high concentrations in the wood, textile and paper industries as well as in tobacco smoke, smog and combustion engine exhaust. In general, three aldehydes (formaldehyde, acetaldehyde and acrolein) are under special observation regarding their adverse health effects [110]. Formaldehyde is a simple molecule and as such has only a few transitions in the THz frequency range. Schmalz et al. observed only one transition for formaldehyde in the frequency range 491–498 GHz, whereas acetaldehyde (CH_3_CHO) showed several absorption lines, 150 and 109 in the frequency range 238–252 GHz and 491–498 GHz, respectively [7]. 

**Figure 8:**
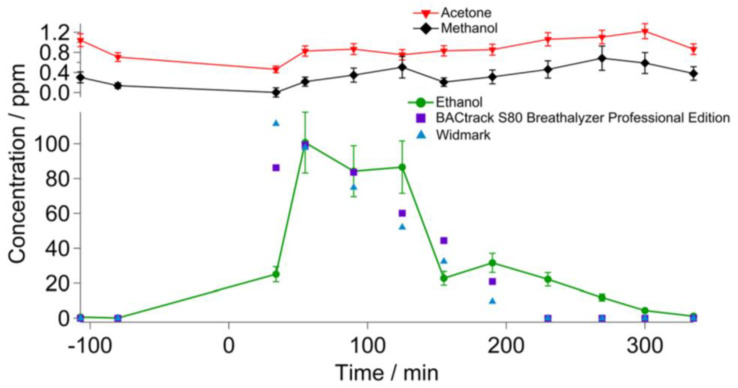
Comparison of ethanol, methanol and acetone concentrations in exhaled breath measured by THz spectroscopic method and ethanol concentrations measured by a commercial BACtrack breathalyser (values indicated by purple squares in the figure). Blue triangles indicate values calculated using Widmark’s formula, which considers blood alcohol content as a function of the subject’s body weight, gender, the amount of alcohol consumed and time passed after the start of alcohol intake. The horizontal scale represents the time since the start of alcohol consumption. Reprinted from [47], with the permission of AIP Publishing.

Ethanol, methanol and acetone are also biomarkers for several diseases that were analysed by THz sensing [47]. Acetone in breath is a marker for non-insulin diabetes [90]. Ethanol, methanol and acetone were also detected in the exhaled breath of a person who consumed alcohol [47], thus the THz gas sensor can be an alternative to a commercial breath alcohol tester in terms of sensitivity, sample volume and specificity (Figure 8). The measured and calculated sensitivities for ethanol, methanol and acetone were 75 ppb, 45 ppb and 18 ppb, respectively. These results are comparable to laser-based breath analysers and mass spectroscopy-based techniques [47].

#### 3.3.2. Ammonia

Jacobsen et al. demonstrated a chemical recognition system which was able to discriminate between NH_3_- and H_2_O-dominated phases in a binary mixture by estimating the partial pressures of components, although the resonances at 0.572 and 1.168 THz for NH_3_ are close to the resonances of H_2_O at 0.557 and 1.163 THz [44]. Almost the same but more precise rotation frequencies of NH_3_ (0.572498 THz) and H_2_O (0.55693 THz) were observed by Sun et al. at pressures well below 10 Pa [101]. Harde et al. performed THz-TDS measurements on ammonia vapour at room temperature to experimentally and theoretically study the absorption and dispersion of the selected gas species, which is important in astrophysics [111]. Besides 50 spectral lines of ground state observed above 600 GHz, an additional broad absorption band around 1.6 THz was noticed that most probably originates from ammonia dimers ((NH_3_)_2_). The THz spectrum of ammonia at the gas concentration of 100 ppm was also measured by a CW-THz system by Hepp et al. [9]. Considering the SNR, the detection limit was reduced to approximately 20 ppm at a pressure of 100 Pa. THz ammonia sensing can also be used for medical diagnostics since ammonia in a urease breath test is a biomarker for heliobacteriosis and carcinoma of the lung [90].

#### 3.3.3. Aromatic Hydrocarbons

Aromatic hydrocarbons are toxic environmental pollutants found in the soil, groundwater and air. Their origin in the environment arises from natural and anthropogenic activities. Both PAHs and nitropolycyclic aromatic hydrocarbons (HPAHs) have long been of interest in the fields of environmental science due to their toxic effects on human beings. PAHs are released into the environment by incomplete combustion of organic compounds during industrial processes or other anthropogenic activities [112]. PAHs are compounds with a ring structure, where two or more benzene rings are fused and/or in various configurations of merged pentacyclic molecules. NPAHs are formed in the atmosphere by reactions between nitrogen oxides and hydroxyl radicals with the parent PAHs [113,114].

For both types of chemical compounds in a solid state, naphthalene as a PAH and 1-nitronaphtalene as an NPAH, Du et al. measured THz spectra and found a significant difference between them in the frequency range of 0.1–2.2 THz [114]. The visible change in absorption intensity most probably originates from the difference in molecular structure and lattice vibration modes. The absorption coefficient of NPAH is much stronger in comparison to PAH. 1-nitronaphtalene exhibited specific absorption peaks at frequencies of 0.52, 0.71, 1.05, 1.26, 1.35 and 1.75 THz, whereas for naphthalene no absorption peaks were observed below 2 THz. The reason is probably the nitro group bonded on the aromatic ring. Cataldo et al. also studied more than 30 different PAHs in crystalline form that are expected to be found in a gaseous phase within the interstellar medium by THz spectroscopy in the spectral range between 600 and 50 cm^−1^ for astrochemical purposes [115]. The results are included in a wide database and can be of interest in other branches of chemistry or other applications, including pollutant sensing. Han et al. also analysed solid samples of PAHs by THz-TDS [116].

#### 3.3.4. Carbon Oxides

Although carbon dioxide (CO_2_) is an essential ingredient in photosynthesis, it is also one of the greenhouse gases that contribute to the warming of the Earth’s atmosphere. Its level in the atmosphere significantly increased with industrial development. The main CO_2_ sources are associated with motor vehicles, aircraft, power plants and other activities that involve the burning of fossil fuels. CO_2_ is also one of the indoor air pollutants that are responsible for respiratory health symptoms [117]. 

Allodi et al. measured the THz spectra for CO_2_ ice samples and were unable to observe any features corresponding to pure CO_2_ ice in the 0.3–7.5 THz spectral range [118]. However, they observed the impact of CO_2_ molecules on the THz spectrum of water where the resonances of H_2_O ice were attenuated, distorted and/or shifted when contaminated with CO_2_. Thus, THz spectroscopy plays a remarkable role in its use in the chemistry of the interstellar medium and the historical studies of thermal transformations within the ice. 

While CO_2_ absorption is very weak in the THz region, incomplete combustion can be monitored by THz spectroscopy via other produced gases such as CO, which consists of polar molecules and shows THz spectral signatures [39]. Hu et al. demonstrated that CO has many distinct characteristic absorption peaks between 0.2 and 2.5 THz [11]. Moreover, Uno and Tabata measured CO with 99.9% purity with THz-TDS and they found 12 rotational transition frequencies in the frequency range from 0.2–2.0 THz [61]. They also observed two peak shoulders at 558 GHz and 991 GHz while burning woodchips, which could belong to CO. A very similar experiment was performed by Shimizu et al., who measured the absorption spectra of yellowish smoke produced during heating moulded charcoal [119]. The THz spectrum showed an absorption line around 692 GHz, which agrees with the absorption line of CO and was confirmed by increasing the mass of moulded charcoal which increased the intensity of this peak. Another absorption peak was observed at 921 GHz. In a higher frequency range between 1 THz and 2.5 THz, the visibility of spectral peaks for CO is reduced, thus, Su et al. demonstrated that some chemometrics methods like empirical mode decomposition (EMD) can improve the visibility necessary for gas recognition [120]. Kilcullen et al. carried out experimental and theoretical studies on commensurate echoes emitted from CO by using THz-TDS [87]. They demonstrated that a linear dispersion model of THz wave propagation through a gas sample accurately predicts the shape of the echoes emitted from the same sample. The demonstrated model included the rotational constant of the CO molecule and the average self-pressure-broadening parameter. Furthermore, the self-pressure broadening of CO was determined by fitting the model parameters to experimental results through a range of pressures in the time domain. Thus, the characteristic relaxation time can also be obtained without the need to resolve spectral linewidths in the frequency domain. In addition, CO is also related to asthma and respiratory infection. Therefore, it was also analysed as a biomarker in exhaled breath analysis by THz spectroscopy [90].

#### 3.3.5. Chlorides

Hydrogen chloride is a colourless gas that is highly corrosive and toxic. Since it is readily dissolved in water, it also reacts with water molecules in the air to give clouds of hydrochloric acid that is easily washed out by rain and moisture. Therefore, hydrogen chloride pollution is a global as well as local environmental problem. The most significant releases of HCl occur in coal-fired power stations and waste burning (e.g., polyvinyl chloride (PVC)). Shimizu et al. measured smoke produced from PVC [119]. The absorption spectrum of the smoke showed a spectral peak at 626 GHz, which coincides with the absorption line of HCl. By using a CW-THz gas analysis system, Hepp et al. found the optimised range to be from 1.1 THz to 1.3 THz, where HCl showed the strongest absorption lines [9]. The gas phase of HCl was also recognised as a single species in a pressure range from 0.3 to 13 kPa by using a correlation type of analysis in the time domain [44]. HCl is also present in the Earth’s atmosphere in significant amounts, therefore the accurate determination of its rotational constants is convenient for the frequency calibration of atmospheric spectra. A series of rotational transitions between 0.3 and 6 THz for HCl was already measured by Nolt et al. more than thirty years ago [121]. 

Harmon and Cheville performed THz measurements in the time domain using a 5.0 m path length at pressures down to 1 Pa to detect methyl chloride (CH_3_Cl) in the low ppm range in near real time [122]. Using THz radiation, the rotational constants of the two methyl chloride isotopes were determined by Harde et al. [123]. The index of refraction and the absorption coefficient for CH_3_Cl were also determined through the adsorption of gas molecules on hydrophilic aerogel [91].

#### 3.3.6. Monoatomic Gases

Sang and Jeon measured pressure-dependent refractive indices of monoatomic gases such as helium (He), argon (Ar) and krypton (Kr) in the THz frequency range [88]. They observed that the refractive indices of these gases scaled linearly with pressure at constant room temperature. Furthermore, they noticed that the refractive indices are strongly determined by the polarisability of the gas molecules. The molecules of heavy noble gases (Kr, Ar and Xe) are usually trapped in air from ice cores, which can be used to reconstruct past mean ocean temperatures [124]. As mentioned earlier, the absorption of THz waves is very sensitive to polar molecules such as water, but the THz transmittance of ice is much higher than that of liquid water [54], therefore a THz system can also be applied for the detection of gases caught in ice.

#### 3.3.7. Cyanides and Nitriles

Nitriles are organic compounds with a –C≡N functional group, whereas cyanides are inorganic compounds containing the C≡N− ion. The major sources of cyanides are mining, galvanic and chemical industries as well as tobacco smoke [125]. In air, cyanide ions are present mainly as HCN and can be transported over long distances from the emission source. Because cyanide ions have a toxic effect on human health, it is necessary to determine their concentration directly in the atmosphere or representative samples taken from the environment.

Some cyanides and nitriles have already been analysed by THz spectroscopy. For instance, HCN showed spectral lines every 88 GHz [84]. Besides CO, Shimizu et al. observed characteristic peaks of HCN at 709, 798 and 887 GHz while measuring the smoke emitted from moulded charcoal [119]. Hindle et al. determined a HCN concentration of 210.3 ppm within a cigarette smoke mixture [58]. The photon energies of molecules are important for their detection in the THz range, but gas detection at low concentrations is difficult due to the weak dipole moments of the gas at THz frequencies. Qin et al. have developed a unique THz-based sensor structure from a porous core of photonic crystal fibres with high sensitivity for gas detection [37]. Using the developed sensor, they demonstrated the detection of the toxic gas hydrogen cyanide based on its resonant frequency, which can be detected at a low concentration of 2 ppm below normal atmospheric pressure (1 atm). Such an approach can be used to detect toxic gases in air pollution monitoring.

Methyl cyanide (CH_3_CN), also known as acetonitrile, has lines every 18 GHz [84]. Hsieh et al. performed static and dynamic measurements of CH_3_CN samples with various concentrations [39]. Differences among THz spectra in static measurements showed that they tested samples with various concentrations. The gas concentrations for each sample at atmospheric pressure were further determined by the proposed theoretical model. The lowest determined concentration was 0.341%, equivalent to the 20 ppm detection limit. In dynamic measurements, they measured THz spectra of CH_3_CN mixed with smoke at atmospheric pressure. In the experiment, they monitored in real time the volatilisation of CH_3_CN by observing the temporal change in the THz power spectrum concerning elapsed time. The characteristic spectral features of CH_3_CN were easily distinguished after 30 s. The detection limit in dynamic operation was 200 ppm. The spectral features of HCN and CH_3_CN were also identified in the THz spectra of smoke emitted from the incomplete combustion of nylon fabric by a CW-THz system [126]. 

Also, other cyanides were analysed in the THz range. Propionitrile, also known as ethyl cyanide (C_2_H_5_CN), exhibits spectral lines every 9 GHz [84], whereas acrylonitrile (CH_2_CHCN) as a biomarker for smokers showed several absorption lines, 439 and 545 in the frequency range of 238–252 GHz and 491–498 GHz, respectively [7]. A high-resolution THz spectrometer based on semiconductor superlattice multipliers has been used to investigate the dynamics of urine vapour composition in cancer patients treated with chemotherapy [45]. THz analysis of urine samples detected a set of nitriles that appeared after chemotherapy or increased in content, suggesting that nitriles are candidates for biomarkers of chemotherapy nephrotoxicity at an early stage of treatment and are indicative of organ damage. The proposed THz system operates in four different frequency ranges and allows the assessment of most substances in the fluid vapour, avoiding the strong absorption lines of substances such as water and ammonia, which may otherwise obscure the detection of target metabolites.

Sitnikov et al. developed a THz-TDS system allowing remote open-path gas detecting and identifying gases in the presence of water vapour absorption lines [127]. Acetonitrile vapour was detected and successfully evaluated. An organic crystal with 2% conversion efficiency pumped by femtosecond laser pulses in the NIR range was used to generate THz radiation. The use of a new high-power broadband terahertz (0.2–3 THz) source opens new possibilities for further increasing the open-space distances in remote sensing. 

#### 3.3.8. Nitrogen Oxides

With industrial development, the global atmospheric concentration of N_2_O is continuously rising, thus causing changes to ozone concentrations, increased atmospheric greenhouse gas effects and acid rain formation [128]. Although its atmospheric concentration is only 320 ppb, some authors claimed that N_2_O has an even worse influence on the greenhouse effect than CO_2_ due to its greater ability to absorb IR radiation [129]. N_2_O as an important trace gas in the atmosphere exhibits spectral features in the THz frequency range, which can be useful as a monitoring tool for remote and in situ measurements. The characteristic spectral pattern of N_2_O occurs every 25.1 GHz and reflects one strong ground state transition, two vibrational transitions and three primary isotopic transitions [130]. The remote sensing of N_2_O gas with a 64% concentration at a distance of 1.3 m in the frequency range between 200 and 500 GHz showed two characteristic spectral peaks at 452 and 478 GHz. Similar results were obtained for N_2_O at a distance of 3.6 m. In one experiment, Kim et al. extended the beam path to 18.61 m using several mirrors in a gas cell. Due to the long THz beam path, a gas concentration of N_2_O as low as 1.0% was enough to measure the absorption coefficient [131]. A gas cell with a longer THz beam path would be needed to measure gas concentrations below 1%, since Beer’s law that absorption is inversely proportional to gas concentration and beam path applies. Thus, the active THz system developed by Shimizu et al. proved that remote gas sensing is possible by THz spectroscopy [8]. In addition, N_2_O is also used for the calibration of laboratory spectroscopic systems in the IR region [130,132].

Nitric oxide (NO) is an insignificant constituent of our atmosphere which importantly contributes to the catalytic decomposition of ozone. Nitric oxide is also of interest for molecular spectroscopy studies because it is the only stable diatomic molecule with an odd number of electrons [133]. Additionally, nitric oxide detected at a frequency of 150.176 GHz in breath is a marker for asthma, bronchiectasis and other lung diseases [90]. The measurements also showed the increased concentration of NO in the exhaled breath of lung cancer patients against healthy patients.

Nitrogen dioxide (NO_2_) is an important air pollutant because it contributes to the formation of photochemical smog and causes respiratory problems. Its major source is the burning of fossil fuels. THz spectroscopy is capable of detecting NO_2_ since it showed a characteristic absorption line at 493.28 GHz [7].

#### 3.3.9. Ozone

Ozone (O_3_) as a so-called secondary air pollutant is a product of various chemical reactions under the Sun’s influence involving precursors such as nitrogen oxides and volatile organic compounds (VOCs). The destruction of stratospheric ozone that leads to higher UV light intensities is an air pollution problem with global dimensions. The molecules of ozone are destroyed in cyclic chemical reactions by chlorine, bromide, NO and OH radicals. The presence of ozone is changing the chemical composition of the atmosphere and affects not only human health but also biological evaluation and climatological imbalances. Slanina reported that the ozone concentrations across Europe slowly increased at a rate of 1–2% per year, from 10 ppb to over 50 ppb [1]. Remote sensing of ozone is performed mainly in the UV and the mid-IR region.

Since ozone molecules absorb THz radiation, remote sensing by spectroscopic techniques at THz frequencies is of special interest. Drouin et al. determined the ozone mixing ratio (a mixture of O_3_ and O_2_) by measuring seven pure rotation ozone lines in the frequency range from 692 to 779 THz [134]. Furthermore, the TErahertz and submillimeter LImb Sounder (TELIS) spectrometer [135] measures the ozone concentration in the stratosphere and the chemistry of chlorine and bromine, which are responsible for the degradation of ozone molecules.

#### 3.3.10. Particulate Matter

Economic growth and technological development contribute to increasing problems of air pollution with particulate matter in many countries. Fine particulate matter in the atmosphere with particles below 2.5 microns in size (PM_2.5_) harms human health and climate conditions [136]. PM_2.5_ consists of metallic elements, inorganic compounds, trace elements and organic compounds [136,137]. Moreover, organic and nitrogen oxide emissions significantly contribute to ozone formation [51]. The World Health Organization (WHO) has, therefore, set the air quality guidelines and each country has defined environmental limits for the mass concentration of PM_2.5_ [136]. Determination of PM_2.5_ is usually performed by a standard method using a membrane filter known as the Federal Reference Method (FRM) [138], where first particles are separated by their size and then their weight is determined by estimating the change in membrane weight. 

In China, researchers demonstrated that THz waves can be used as a tool to monitor and inspect the PM_2.5_ concentration in the frequency range from 2–10 THz [136]. The samples were collected by an air sampler, where the particles were first separated by a particle size separator to PM_10_ and PM_2.5_. The PM_2.5_ particles were further collected on a membrane made of quartz. During the THz measurements of sample membranes with various concentrations, an empty membrane was used as a reference. For the selected site, the THz spectrum showed three spectral features at 4.4, 6.0 and 7.0 THz. The spectral features differ from city to city since the atmospheric pollutant constitution is changing [137] due to industry, traffic, urbanisation, natural processes, weather conditions and other human activities. However, for PM_2.5_ two distinct absorption bands exist between 2.5 and 7.5 THz [51]. By increasing the concentration of the PM_2.5_ on a membrane, the absorbance of THz radiation also increased [136]. By using a selected single-frequency or distinct absorption band in the THz frequency range, the data acquisition time can be improved, which can give a new opportunity to THz systems to become a real-time tool for estimation of PM_2.5_ concentrations, to investigate the composition of a particulate pollutant or to identify different pollution sources.

Another pollution source that is related to PM_2.5_ particles is dust, which often arises from dust storms or pollution at construction sites. The dominant constituents in the dusty environment are metallic oxides or water-soluble ions like Al, Fe and Ca. The metallic oxides show spectral features in the range from 2.5 to 7.5 THz [51]. For THz characterisation of metallic oxides, anion and cation evaluations are important. Of all elements, only an oxygen atom can exist in an anion state. Because the anion O^2-^ can be in conjunction with many other elements, it contributes to a strong vibration at approximately 3.36 THz. The cations contribute to strong vibrational modes at higher THz frequencies at around 5.84 and 6.91 THz [51]. By using special mathematical methods such as two-dimensional correlation spectroscopy (DCOS) the overlapping of spectral features of the constituents can be resolved.

#### 3.3.11. Sulphides

Hydrogen sulphide is a colourless and toxic gas, which poisons the nervous system and other organs. It is emitted in the gaseous state by various industries such as petroleum refining, wastewater treatment plants (WWTPs), biogas extraction and paper production [58]. Its odour is unpleasant for a residential area and a long exposure to high concentrations is toxic for workers [139]. Therefore, controlling the existence and concentrations of H_2_S in the air is important to reduce the toxic levels of the gas, which can be reduced in time by appropriate removal actions to ensure safe environmental conditions for WWTP workers and other neighbouring residents. Furthermore, H_2_S is also produced through the anaerobic digestion of organic material in biogas extraction. High concentrations of H_2_S reduce biogas quality, cause corrosion of concrete and steel parts of the production process and further contribute to emissions of sulphur dioxide during combustion [140].

The H_2_S molecule is an ideal candidate for THz evaluation since it is asymmetric and has a permanent dipole moment. It exhibits many intense rotational transitions in the THz frequency range. The transitions can be easily attributed to sulphur isotopes present in the H_2_S gas [58]. Cai et al. determined the equivalent interval between adjacent absorption peaks for H_2_S in the frequency range from 0.2–2.6 THz, which is approximately 0.26 THz [104]. THz spectroscopy was also applied to detect H_2_S as a biomarker of diabetes patients in exhaled breath analysis at the frequency of 168.762 GHz [90].

Carbonyl sulphide is the most abundant tropospheric pollutant with a simple molecule that has only a few transitions in the THz frequency range. Schmalz et al. observed only one transition in the frequency range 238–252 GHz [7]. For its THz spectral analysis, a spectrometer with a spectral resolution of 1 MHz is required at pressures in excess of 300 Pa where the spectral line broadening is dominated by molecular collisions [58]. In addition, Bigourd et al. demonstrated on OCS that spectroscopic parameters such as the rotational constants, centrifugal distortion and relaxation times may be evaluated from THz data in the time domain [102]. Characterisation in the THz range is also interesting for astrophysics as it was detected in the atmosphere of Venus and the interstellar medium [58,141].

#### 3.3.12. Sulphur Oxides

Sulphur monoxide (SO) is interesting to study from the astrophysical point of view as well as in atmospheric chemistry since it is an important reaction intermediate in combustion processes. The pure rotational transitions of SO were already observed in the THz region in 1994 by Cazzoli et al. [142]. For instance, in one study, 102 rotational transitions were recorded for SO in the frequency range of 1.3–2.8 THz [143]. Several spectroscopic studies have also been performed on SO isotopes [144] where the two most abundant isotopologues, ^34^SO and ^33^SO, showed 48 transitions up to 1.388 THz and 21 transitions up to 978 GHz, respectively. 

The sulphur dioxide (SO_2_) present in the atmosphere can originate from natural and artificial sources (volcanoes, traffic, industry, oil refineries). The THz spectrum of SO_2_ at the gas concentration of 500 ppm was measured by a CW system by Hepp et al. [9]. Considering the SNR, the detection limit was reduced to approximately 100 ppm at a pressure of 100 Pa. Cai et al. determined the equivalent interval between adjacent absorption peaks in the frequency range from 0.2–2.6 THz which is approximately 0.11 THz [104]. The measured value was in good agreement with the calculations of rotational energies and transition frequencies based on quantum mechanics. Voitsekhovskaya and Egorov calculated the spectral lines of SO_2_ molecules in the frequency range from 0.1 to 10 THz [94]. They also estimated the absorption coefficients at different temperatures (300–1200 K) in the same frequency range and analysed the contribution of each state to the absorption coefficient. At low temperatures, the absorption coefficient reflects the rotational transitions within the ground state, whereas at higher temperatures rotational transitions of other states dominate. All calculated results are summarised within the database of SO_2_ vibrational–rotational line parameters for different temperatures and are ready for use in further gas spectral simulations and determination of gas concentrations.

#### 3.3.13. Volatile Organic Compounds

Volatile organic compounds are extremely toxic carbon-based compounds with a high vapour pressure at room temperature and as such importantly contribute to regional atmospheric pollution. The quantitative and qualitative analysis of VOCs is necessary for various applications, including medicine, security and environmental monitoring [86]. Within this subsection, VOCs as a general group of chemicals are given, whereas some individual VOCs are included in the subsequent subsections.

VOCs exhibit dense spectra in the THz frequency range. Within the Doppler limit and at low pressure, there is almost no overlapping of spectral lines. Therefore, the VOCs can be detected and identified even within a small spectral band with the THz system of sufficient spectral resolution. With increasing pressure, the identification is usually lost due to spectral line broadening and overlapping [22]. For VOC spectral analysis, the ideal THz system is capable of simultaneous detection of several substances mainly because in real applications, like human breath analysis or the detection of escaping gases at accidental sites, a mixture of gases and their reaction products are present [22].

The most used method to remove VOCs from the environment is adsorption by activated carbon due to its large specific surface area and large pore volume [107,145,146]. Therefore, also monitoring the kinetics of adsorption processes is crucial to detect and identify VOCs. The kinetic process of absorption is in general described by theoretical models, such as the adsorption reaction model and adsorption diffusion model. Zhu et al. applied THz spectroscopy to characterise the adsorption dynamics by the pseudo-second-order kinetic model and the relationship between the THz signal peak amplitudes versus the adsorption time [107]. This model showed the proportional relationship between the adsorbed amount of VOC and the THz signal peak amplitude. The kinetic process was monitored by measuring the THz spectra of VOCs dropped on active carbon fibre cloth at room temperature. During the monitoring, the THz power increased in different frequency ranges of the THz spectrum due to the volatilisation, diffusion and adsorption of VOCs on the surface of activated carbon. Zhu et al. also monitored the adsorption efficiency of VOCs by estimating the diffusion rate constant, which can be defined by the THz double exponential model [147]. In comparison to the pseudo-second-order model, this model also considers the diffusion process, which is divided into two steps. The rapid step involves external and internal diffusion where VOCs adhere to the external surface of the adsorbent. The following slow step describes an interparticle diffusion, where the VOC molecules penetrate the inner layers of activated carbon. The absorption rate also depends on the molecule size. Bigger molecules require more time to enter inner holes and to be absorbed. For example, the absorption rate of ethanol is higher than that of butyl acetate. Besides activated carbon, polymer microporous membrane was also demonstrated as a potential candidate for the adsorption of VOCs in the THz regime [148]. A multilayer-stacked microporous polymer structure was successfully used in ambient atmosphere and at room temperature to sense different types of VOCs with different dipole moments and to discriminate various concentrations of organic vapours at THz frequencies.

VOC sensing is especially important for medical purposes because human breath contains more than 3500 trace gases [149], which are related to several diseases or exposure to environmental pollutants and toxic industrial chemicals [22,90]. Thus, THz spectroscopy can play a key role in safe and fast medical diagnostics. Several VOCs (e.g., (CH_3_)_2_CHOH, (CH_3_)_2_CO, CH_3_OH, OCS, CH_2_CHCN, CH_3_CHO, H_2_CO) classified as medical breath biomarkers exhibit strong absorption lines around 245 and 500 GHz [86]. Schmalz et al. demonstrated that a frequency range from 230–260 THz allows the detection of a large number of VOCs with strong absorption and adequate absorption lines [7]. THz-FDS has proven to be a reliable methodological approach with high resolution in gas detection and quantitative evaluation of VOCs in multicomponent mixtures, which is useful for human breath analysis and environmental/occupational monitoring [41]. Recently, some new systems have also been developed, such as THz-TDS with ceramic architecture, which are used to create unique crystal structures, morphologies and properties [150]. This system allows the selective detection of gas in a mixture of VOCs at ppm concentrations by controlling the architecture of porous glass coated with ceramic films on which the VOC is adsorbed. Each VOC is separated due to a “surface modification” effect. Since THz sensing can simultaneously detect and identify several substances, it is a candidate for non-invasive exhaled breath analysis besides mass spectrometry, IR spectroscopic sensors, electrochemical sensors and chemiluminescence [47,90]. Since exhaled breath contains water vapour that causes spectrum broadening, a preconcentrator is necessary to reduce the water concentration by a factor of several hundred and to enhance the sensitivity for gas detection.

Vaks et al. are pioneers in using THz gas spectroscopy for the analysis of the composition of products of thermal decomposition from different human body fluids, including blood, urine and saliva, and tissues [45,46,90,151]. The most intense absorption lines in the rotational spectrum of molecules, including organic compounds, are in the THz frequency range. For this reason, this approach is suitable for the study of multicomponent gas mixtures of different origins, including biological ones, formed during thermal decomposition of tissues or body fluids. By detecting absorption lines in spectra obtained during the transmission of radiation through a sample that is a multicomponent gas mixture, it is possible to identify differences in the composition of samples corresponding to different pathologies and diseases. High-resolution THz spectroscopy has thus been used to investigate the composition of metabolites in the thermal degradation products of healthy tissue and body fluid. In one of our previous reviews, we summarised the potential of THz spectroscopy to analyse different urinary metabolic biomarkers for diagnosis of cancer [21].

**Table 2:** Experiments performed by THz spectroscopy for gas analysis included in the review.

References	Gas Sample	THz System	Frequency Range (THz)	Study
[102]	OCS	THz-TDS	0.1–1.5	Determining the rotational constant, centrifugal distortion and relaxation times
[104]	SO_2_, H_2_S	THz-TDS	0.2–2.6	Spectral peak analysis
[134]	O_3_	FTIR	0.67–0.77	Determining ozone concentration
[114]	C_10_H_8_, C_10_H_7_NO_2_	THz-TDS	0.1–2.2	Measuring absorption spectra for PAHs and NPAHs
[47]	CH_3_OH, C_2_H_5_OH, (CH_3_)_2_CO	CW-THz	0.21–0.27	Detection of gases within the exhaled breath of a person who consumed alcohol
[109]	C_3_H_8_O	THz-TDS	0.2–3.5	Detection of gas concentration in the range of 50–500 ppm
[111]	NH_3_	THz-TDS	0.08–2.5	Studying the absorption and dispersion of gas
[122]	CH_3_Cl	THz-TDS	0.1–1.8	Detecting gas species in the low parts-per-million range in near real time
[9]	NH_3_, SO_2_	CW-THz	1.1–1.3	Detection limit improvement
[58]	Cigarette smoke, HCN, CO, H_2_O, H_2_CO	CW-THz	0.6–2.3	Measurement of multiple species in one sample
[58]	H_2_S	CW-THz	1.016–1.028	Spectrum measuring, sulphur isotope evaluation
[37]	HCN	CW-THz	1.2399	Low-concentration detection of toxic gases
[39]	CH_3_CN	ASOPS-THz-TDS	0.2–1	Static and dynamic study of gas concentrations in a smoky environment
[127]	CH_3_CN	THz-TDS	0.2–3.0	One-path remote gas detection, quantification and recognition
[11]	CO	THz-TDS	0.2–2.5	Spectral analysis
[87]	CO	THz-TDS	0–3.3	Direct measuring of commensurate echoes from gas molecules
[136]	PM_2.5_	THz-TDS	0–10	Studying the concentration of PM_2.5_ in air
[137]	PM_2.5_	FTIR	2–8	Studying the composition and concentration of PM_2.5_ in two different geographical areas
[144]	SO isotopes	CW-THz	0.3–3.3 (tunable)	Studying the absorption spectra of gas isotopes
[141]	OCS	CW-THz	0.1–2.0	Transition characterisation, self-broadening coefficient determination, analysing dependency on pressure
[99]	H_2_O (vapour)	CW-THz with disc microresonator	0.4–0.65	Detection of water vapour at a concentration of 4 parts per million in the atmosphere
[133]	NO	Evenson-type tunable FIR spectrometer	0.99–4.75	Determining transitions around 2 THz for better prediction of higher rotational states
[22]	VOCs	THz-FDS	0.238–0.252	Studying the absorption spectra of VOCs as medical biomarkers with several MVA techniques for substance detection and identification purposes
[86]	VOCs	CW-THz	0.245 and 0.5	Spectrum analysis
[88]	He, Kr, Ar	THz-TDS	0.3–4.5	Determining pressure-dependent refractive indices
[7]	VOCs	CW-THz	0.494–0.500	Studying the gas sensitivity for the THz system
[41]	VOCs: methanol, ethanol, isopropanol, 1-butanol and 2-butanol	THz-FDS	0.06–1.2	Determining molar absorption coefficient of VOCs, optical behaviour of VOC/air mixtures
[8]	N_2_O	THz-TDS	0.2–0.5	Remote spectral sensing at 1.3 and 3.6 m
[131]	N_2_O	THz-TDS	0.2–1.2	Absorbance and absorption coefficient measurements using long propagation path
[126]	HCN, CH_3_CN	CW-THz	0.2–0.5	Detection of gases from heated nylon fabric
[119]	HCl, CO, HCN	CW-THz	0.50–0.95	Detection of gases from heated PVC and moulded charcoal
[120]	H_2_O, CO	THz-TDS	0–2.2	THz signal changes with path length in ambient air due to water vapour
[101]	NH_3_, H_2_O	THz-TDS	0.4–2	Gas detection
[61]	CO	THz-TDS	0.2–3	Studying the transmission properties of gases contaminated by aerosols and water vapour
[90,152,153]	(CH_3_)_2_CO, CH_3_OH, C_2_H_5_OH, NH_3_, H_2_S, NO	THz-FDS	0.118–0.178	Detection of gases as disease biomarkers within exhaled breath, tissue and body fluids
[51]	PM_2.5_	FTIR	2.5–7.5	Studying the elemental composition and quantitation of PM_2.5_
[107,147]	VOCs	THz-TDS	0.1–1.6	Monitoring the adsorption process of VOCs
[80]	VOCs	tailor-made THz system	238–252 GHz	Qualitative and quantitative analysis of absorption spectra of gas mixtures measured at different pressures, using independent component analysis (ICA) to predict their concentrations
[150]	VOCs	THz-TDS with ceramic architecture	0.2–1.8	Detection of VOC mixture with ppm-order concentrations
[148]	VOCs	THz-TDS	0.1–0.45	Discriminating between different concentrations of VOC

## 4. Future of THz Technology in the Field of Environmental and Biomedical Applications

THz sensors have advantages and disadvantages with much room for improvement, further development and optimisation for a particular application. However, several obstacles remain to be overcome to enable the successful transition of the THz sensors into real-time environmental applications. Among them, the most important are enhancing the dynamic range of the THz systems, minimising the size of the systems, developing more sensitive THz sensors, enabling high-speed data acquisition and creating an extended database for various gases and other atmospheric pollutants in the THz frequency range.

**Figure 9:**
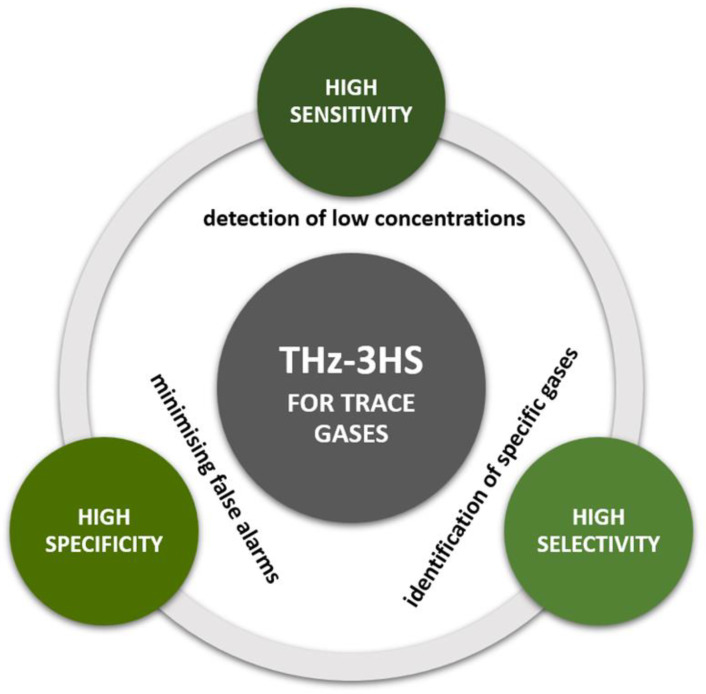
Three parameters (3HS) defining the ability of the THz spectrometer for trace gas sensing.

A THz spectral analyser for trace gas sensing must provide three high parameters (Figure 9). We refer to these parameters as 3HS: high sensitivity, high selectivity and high specificity. All three parameters ensure the high reliability and accuracy of THz measurements [7,90]. The high sensitivity allows THz spectroscopy to detect trace gases in the sample at very low concentrations. High selectivity ensures gas identification, i.e., it can distinguish between different gases in a mixture, which is particularly important for gases with overlapping spectral characteristics. The high specificity ensures that the THz system responds accurately only to the presence of the gas being tested. This means that there is less chance of obtaining incorrect results due to interference from other substances. With the appropriate combination of these parameters, a 3HS THz spectrometer can be a candidate for the next “electronic nose” enabling exhaled breath diagnostics or toxic vapour analysis.

A THz system must ensure reliable detection of trace gases with a concentration between 100 parts per trillion (ppt) and 1 part per million (ppm) in a gas mixture [90]. The real-time detection and identification of trace gases in various atmospheric conditions demand a high SNR to resolve the characteristic spectral features of individual gas species and high-resolution THz measurements to enlarge low-intensity spectral features, which usually disappear within the signal background. High resolution can only be achieved with relatively high-power sources. Thus, further progress is expected in the development of appropriate THz sources for THz-TDS to increase the SNR above 50–60 dB and frequency resolution below 1.5 GHz [108]. Currently, these parameters can be achieved with asynchronous optical sampling [154] or high-speed optical delay using photoconductive antennas [155,156]. SNR is not only dependent on the THz system but also depends on gas properties. Higher concentrations of gas molecules increase the SNR, but the rate of increase is dependent on the type of gas molecule due to their different dipole moment strengths [108]. In general, high SNR can be achieved with a single measurement at high power or by averaging several measurements. The last approach requires a longer measurement time, which is not desirable for gas trace sensing due to the fast changes in environmental conditions and system drift. Usually, a THz system with a frequency resolution lower than 4 GHz is enough to resolve individual spectral lines and discriminate between gases in a multigas mixture in ambient conditions [108]. SNR may also increase with a stronger probe beam power that may lead to the detection of gas molecules at the parts per billion (ppb) level and real-time measurements in the future [108]. Therefore, high-frequency resolution should enable spectroscopic measurements of toxic gases even at dangerous sites [9]. In such situations, remote spectral sensing of gases is preferable, ensuring the accurate identification of gas molecules and their concentration from the THz absorption spectra [8].

The sensitivity of THz measurements is usually defined as a ratio between the known concentration in ppm and the SNR of the strongest absorption line [86]. The absorption peak intensities are determined by using least-squares peak modelling, whereas the noise is estimated by calculating the standard deviation in the vicinity of the peaks [9]. The CW-THz system using a gas preconcentrator has achieved sensitivities of up to 3 ppm [86] depending on the selected frequency range, whereas a CW-THz system with only a gas cell [9] achieved a detection limit of approximately 20 ppm. Long propagation paths also yield superior sensitivity [47]. The minimum detectable amount of the substance is given by the ratio of the detector noise equivalent power (NEP) to the source power per unit bandwidth. Therefore, the change in power on the detector is proportional to the absorption coefficient and the length of the absorption cell [7]. The sensitivity can be improved by selecting a source with relatively higher output power [7]. Moreover, the sensitivity and the speed are linked through detector–signal integration time and are sample dependent. The lowest detection limit from reviewed references was mentioned by Neese et al., who achieved a sensitivity of around 2 ppt for deuterium isotopic species by using an absorbent and analysis of several statistically independent data points [84]. In this respect, in Table 3 we summarise the sensitivity of THz measurements as the determination of the minimum concentration of the analyte, i.e., the target gas. This is also referred to as the limit of detection (LOD), which is usually defined for chemical gas sensors. Unfortunately, it is only possible to refer to the values of the LOD parameter for some of the measurements included in this review paper. Most commonly, the sensitivity is expressed in ppm or ppb. For example, a sensitivity for X gas of 1 ppm means that the instrument can detect the presence of X gas at a concentration of 1 part per million (ppm) in the sample. THz spectroscopy is usually very sensitive to low gas concentrations. This means that it is possible to detect the presence of gases in a sample even at low concentrations such as ppm or ppb. The sensitivity to different gases with THz spectroscopy is highly dependent on the specific experimental conditions, selected equipment and target gases. The target gas used for the analysis is crucial, as some gases may strongly absorb THz radiation, while others may show only weak absorption. The sensitivity also depends on the sampling method of the gas itself and the interaction path length. Thus, the sensitivity varies for different gases and different THz frequencies. To improve the detection limit of gases from ppm to ppb levels, THz spectroscopic systems need to be equipped with a chamber, a long gas cell, a preconcentration system or even a heating device.

THz analysis for environmental applications requires a compact and low-cost system for gas spectroscopy. One such system was described by Neuimaier et al. where they demonstrated a solid-state device based on a SiGe transmitter and receiver operating at frequencies around 245 GHz and 500 GHz [86]. To date, the THz systems based on QCLs are currently the only compact sources operating with high output powers above 1 THz [64], thus allowing applications such as trace gas analysis. On the other hand, at disaster sites with cluttered environments, such as fires or industrial chemical spills, where rapid identification and concentration determination are important, a transportable analysing CW-THz system capable of sensing a variety of toxic gases is preferable [9]. For real applications, gas collection and external parameter control have to be considered to achieve the most reliable results [22].

## 5. Conclusions

The present review demonstrates that THz spectroscopy has great potential to become an important analytical tool for the detection and quantification of gas phase and particulate matter atmosphere pollutants. In addition, it offers direct and simultaneous measurements of multicomponent gaseous species even in environments contaminated by aerosols or other particles. Current THz systems allow measurements of several gas species at the ppm sensitivity level if their rotational spectral lines are widely spaced.

Most THz systems still require calibration before measurements are taken to ensure the accuracy of the system and, thus, reliable and accurate gas concentration measurements. The characteristics of the THz system determine whether the selected THz system can be used for a particular type of sample and environment in which the measurement is to be performed (e.g., environmental monitoring, industrial process control, medical diagnostics). When choosing a THz system for gas analysis, one must carefully consider the following:

1. the frequency range that is species dependent and must ensure enough space for gases with sparser spectra;
2. the spectral resolution that ensures distinguishing adjacent peaks that lie close together (higher resolution may be needed for detailed gas analysis);
3. The 3HS parameters to detect lower concentrations of gases in the sample, distinguish between different gases and identify a particular gas;
4. a pressure that should be low enough that individual narrow spectral lines of species in the mixture do not disappear in the Doppler broadening (it demands a vacuum system to evacuate the spectrometer cell to the desired pressure and to pull the gas sample into the preconcentrator tube);
5. an absorption cell which should be long enough to achieve appropriate sensitivity even for small amounts of a sample; and
6. a gas preconcentrator to improve the sensitivity, especially for small gas volumes.

Further miniaturisation, portability and a decrease in the price of THz systems as well as advances in data processing and spectroscopic analysis strategies can enable future THz systems to provide automated qualitative analysis with quantitative results for various gases in complex gaseous mixtures by direct measurement or by using multiple preconcentration strategies. Recent advances in chemometrics have a great potential to further improve the sensitivity of future THz systems so that they can play an important role in future gas sensing, not only in environmental monitoring but also in indoor air quality analysis and medical diagnostics.

## Figures and Tables

**Table 1:** Advantages and disadvantages of TH-TDS and THz-FDS to be considered in gas analysis.

	THz-TDS	THz-FDS
THz source	ultrafast femtosecond laser in combination with non-linear crystals or photoconductive antennas (optical rectification or difference frequency generation), free electron lasers (FELs), quantum cascade lasers (QCLs)	continuous-wave (CW) or tunable THz source (QCLs, backward wave oscillators), photomixing using two laser beams and Schottky diodes, FELs, femtosecond laser
THz detector	electro-optic crystals, photoconductive antennas, bolometers	bolometer, quantum cascade detectors (QCDs), Golay cell, Schottky diodes
Emitted THz radiation	broadband ultrashort THz pulses	frequency-modulated narrowband or continuous-wave THz radiation
Measured data	amplitude and phase as a time-domain analysis of THz waveform	amplitude and phase as a function of the modulation frequency
Spectral resolution	high spectral resolution within wide frequency range	high spectral resolution within the limited frequency range
Spectral information	broadband spectral information	spectral information at specific frequencies
System complexity	complex, more sensitive to environmental parameters	simplified, portable, compact
Trace gas analysis	high sensitivity lower selectivity	lower sensitivity higher selectivity (target-specific gas absorption lines at precise frequencies)

**Table 3:** Sensitivity measurements expressed as the limit of detection (LOD) for various gases in the THz frequency range.

Target Gas	LOD (as Analyte Concentration)	Pressure (kPa)	Temperature (K)	Cell Length (cm)	Reference
n-propanol	50 to 500 ppm	NA	NA	NA	[109]
ethanol	75 ppb (predictions)	101.3	300	200	[47]
methanol	45 ppb (predictions)	101.3	300	200	[47]
acetone	18 ppb (predictions)	101.3	300	200	[47]
ammonia	20 ppm	0.10	NA	500	[9]
acetonitrile	200 ppm	101.3	NA	20 and 50	[39]
hydrogen cyanide	2 ppm	101.3	NA	NA	[37]
sulphur dioxide	100 ppm	0.10	NA	500	[9]
carbon monoxide	40 ppm	13.3–133	300	13.6 cm	[87]
acetonitrile	10 ppm	101.3	300	NA	[108]
water vapour	4 ppm	101.3	300	NA	[99]
dimethyl sulphoxide	50–100 ppm	0.053	297	21.6 cm	[157]
nitrous oxide	1% (10,000 ppm)	101.3	295	1861	[131]
VOC	1 ppm	NA	300	NA	[150]

## Data Availability

The data that support the findings of this study are available from the corresponding author upon reasonable request.

## References

[1] Slanina J. (2007). Air Pollution: The Emission–Effect Relation. Rev. Environ. Sci. Biotechnol..

[2] Wagner T., Beirle S., Deutschmann T., Eigemeier E., Frankenberg C., Grzegorski M., Liu C., Marbach T., Platt U., de Vries M.P. (2008). Monitoring of Atmospheric Trace Gases, Clouds, Aerosols and Surface Properties from UV/Vis/NIR Satellite Instruments. J. Opt. Pure Appl. Opt..

[3] Siegel P.H. THz for Space: The Golden Age. Proceedings of the 2010 IEEE MTT-S International Microwave Symposium.

[4] Liu X., Cheng S., Liu H., Hu S., Zhang D., Ning H. (2012). A Survey on Gas Sensing Technology. Sensors.

[5] Nabiev S.S., Nadezhdinskii A.I., Stavrovskii D.B., Vaks V.L., Domracheva E.G., Pripolzin S.I., Sobakinskaya E.A., Chernyaeva M.B. (2011). Analysis of the Products of the Natural Decay of High Explosives by Subterahertz and Infrared Fourier Spectroscopy. Russ. J. Phys. Chem. A.

[6] Lefferts M.J., Castell M.R. (2015). Vapour Sensing of Explosive Materials. Anal. Methods.

[7] Schmalz K., Rothbart N., Neumaier P.F.X., Borngräber J., Hübers H.W., Kissinger D. (2017). Gas Spectroscopy System for Breath Analysis at Mm-Wave/THz Using SiGe BiCMOS Circuits. IEEE Trans. Microw. Theory Tech..

[8] Shimizu N., Song H.-J., Kado Y., Furuta T., Wakatsuki A., Muramoto Y. (2009). Gas Detection Using Terahertz Waves. NTT Tech. Rev..

[9] Hepp C., Lüttjohann S., Roggenbuck A., Deninger A., Nellen S., Göbel T., Jörger M., Harig R. A Cw-Terahertz Gas Analysis System with ppm Detection Limits. Proceedings of the 2016 41st International Conference on Infrared, Millimeter, and Terahertz waves (IRMMW-THz).

[10] Shigemori T. PT1 Gas Sensors—Status and Future Trends for Safety Applications. Proceedings of the 14th International Meeting on Chemical Sensors (IMCS).

[11] Hu Y., Wang X., Guo L., Zhang C. (2006). Terahertz time-domain spectroscopic study of carbon monoxide. Guang Pu Xue Yu Guang Pu Fen Xi Guang Pu.

[12] van Vuuren P., Lewis I.R., Slater J.B., Tedesco J.M., Fairchild R.C., Human P. (2010). Gas-Phase Raman Spectroscopy—A New Tool in the Process Analysis Toolbox. AIP Conf. Proc..

[13] Petrov D.V., Matrosov I.I. (2016). Spectral Range for Analysis of Natural Gas by Raman Spectroscopy. Proceedings of the 22nd International Symposium on Atmospheric and Ocean Optics: Atmospheric Physics.

[14] Qi R., Yin X., Yang L., Du Z., Liu J., Xu K. (2008). Application of NIR spectroscopy to multiple gas components identification. Guang Pu Xue Yu Guang Pu Fen Xi Guang Pu.

[15] Bacsik Z., Mink J., Keresztury G. (2004). FTIR Spectroscopy of the Atmosphere. I. Principles and Methods. Appl. Spectrosc. Rev..

[16] Abina A., Puc U., Jeglič A., Zidanšek A. (2016). Structural Characterization of Thermal Building Insulation Materials Using Terahertz Spectroscopy and Terahertz Pulsed Imaging. NDT E Int..

[17] Sesek A., Svigelj A., Trontelj J., Widenhorn R., Dupret A. (2015). A Compact THz Imaging System. Image Sensors and Imaging Systems 2015.

[18] Ueno Y., Ajito K. (2008). Analytical Terahertz Spectroscopy. Anal. Sci..

[19] Qiao L., Wang Y., Zhao Z., Chen Z. (2014). Identification and Quantitative Analysis of Chemical Compounds Based on Multiscale Linear Fitting of Terahertz Spectra. Opt. Eng..

[20] Kašalynas I., Venckevičius R., Minkevičius L., Sešek A., Wahaia F., Tamošiūnas V., Voisiat B., Seliuta D., Valušis G., Švigelj A. (2016). Spectroscopic Terahertz Imaging at Room Temperature Employing Microbolometer Terahertz Sensors and Its Application to the Study of Carcinoma Tissues. Sensors.

[21] Abina A., Korošec T., Puc U., Jazbinšek M., Zidanšek A. (2023). Urinary Metabolic Biomarker Profiling for Cancer Diagnosis by Terahertz Spectroscopy: Review and Perspective. Photonics.

[22] Neumaier P.F.-X., Schmalz K., Borngräber J., Wylde R., Hübers H.-W. (2014). Terahertz Gas-Phase Spectroscopy: Chemometrics for Security and Medical Applications. Analyst.

[23] Shen Y.-C. (2011). Terahertz Pulsed Spectroscopy and Imaging for Pharmaceutical Applications: A Review. Int. J. Pharm..

[24] Corsi C., Sizov F. (2014). THz and Security Applications: Detectors, Sources and Associated Electronics for THz Applications.

[25] Solyankin P.M., Nikolaeva I.A., Angeluts A.A., Shipilo D.E., Minaev N.V., Panov N.A., Balakin A.V., Zhu Y., Kosareva O.G., Shkurinov A.P. (2020). THz Generation from Laser-Induced Breakdown in Pressurized Molecular Gases: On the Way to Terahertz Remote Sensing of the Atmospheres of Mars and Venus. New J. Phys..

[26] Mumtaz M., Mahmood A., Khan S.D., Zia M.A., Ahmed M., Ahmad I. (2017). Investigation of Dielectric Properties of Polymers and Their Discrimination Using Terahertz Time-Domain Spectroscopy with Principal Component Analysis. Appl. Spectrosc..

[27] Abina A., Korošec T., Puc U., Zidanšek A. (2023). Review of Bioplastics Characterisation by Terahertz Techniques in the View of Ensuring a Circular Economy. Photonics.

[28] Wietzke S., Jansen C., Reuter M., Jung T., Kraft D., Chatterjee S., Fischer B.M., Koch M. (2011). Terahertz Spectroscopy on Polymers: A Review of Morphological Studies. J. Mol. Struct..

[29] Abina A., Puc U., Zidanšek A. (2022). Challenges and Opportunities of Terahertz Technology in Construction and Demolition Waste Management. J. Environ. Manag..

[30] Wang Q., Wang Q., Yang Z., Wu X., Peng Y. (2022). Quantitative Analysis of Industrial Solid Waste Based on Terahertz Spectroscopy. Photonics.

[31] Nagatsuma T. Terahertz Communications Technologies Based on Photonic and Electronic Approaches. Proceedings of the European Wireless 2012.

[32] Zhang L., Pang X., Pitchappa P. (2023). Editorial for the Special Issue on Broadband Terahertz Devices and Communication Technologies. Micromachines.

[33] Qin J., Ying Y., Xie L. (2013). The Detection of Agricultural Products and Food Using Terahertz Spectroscopy: A Review. Appl. Spectrosc. Rev..

[34] Abina A., Puc U., Jeglič A., Zidanšek A. (2015). Applications of Terahertz Spectroscopy in the Field of Construction and Building Materials. Appl. Spectrosc. Rev..

[35] Cosentino A. (2016). Terahertz and Cultural Heritage Science: Examination of Art and Archaeology. Technologies.

[36] Slocum D.M., Goyette T.M., Giles R.H., Nixon W.E. (2015). Experimental Determination of Terahertz Atmospheric Absorption Parameters. Terahertz Physics, Devices, and Systems IX: Advanced Applications in Industry and Defense.

[37] Qin J., Zhu B., Du Y., Han Z. (2019). Terahertz Detection of Toxic Gas Using a Photonic Crystal Fiber. Opt. Fiber Technol..

[38] Yang L., Guo T., Zhang X., Cao S., Ding X. (2018). Toxic Chemical Compound Detection by Terahertz Spectroscopy: A Review. Rev. Anal. Chem..

[39] Hsieh Y.-D., Nakamura S., Abdelsalam D.G., Minamikawa T., Mizutani Y., Yamamoto H., Iwata T., Hindle F., Yasui T. (2016). Dynamic Terahertz Spectroscopy of Gas Molecules Mixed with Unwanted Aerosol under Atmospheric Pressure Using Fibre-Based Asynchronous-Optical-Sampling Terahertz Time-Domain Spectroscopy. Sci. Rep..

[40] Vaks V., Domracheva E., Sobakinskaya E., Chernyaeva M., Pereira M.F., Shulika O. (2014). Sub-THz Spectroscopy for Security Related Gas Detection. Terahertz and Mid Infrared Radiation: Detection of Explosives and CBRN (Using Terahertz).

[41] D’Arco A., Rocco D., Piamonte Magboo F., Moffa C., Della Ventura G., Marcelli A., Palumbo L., Mattiello L., Lupi S., Petrarca M. (2022). Terahertz Continuous Wave Spectroscopy: A Portable Advanced Method for Atmospheric Gas Sensing. Opt. Express.

[42] Cai H., Wang D., Shen J. (2010). Study of Atmospheric Pollution Using Terahertz Wave. Infrared, Millimeter Wave, and Terahertz Technologies.

[43] Cuisset A., Hindle F., Mouret G., Bocquet R., Bruckhuisen J., Decker J., Pienkina A., Bray C., Fertein É., Boudon V. (2021). Terahertz Rotational Spectroscopy of Greenhouse Gases Using Long Interaction Path-Lengths. Appl. Sci..

[44] Jacobsen R.H., Mittleman D.M., Nuss M.C. (1996). Chemical Recognition of Gases and Gas Mixtures with Terahertz Waves. Opt. Lett..

[45] Vaks V., Anfertev V., Chernyaeva M., Domracheva E., Yablokov A., Maslennikova A., Zhelesnyak A., Baranov A., Schevchenko Y., Pereira M.F. (2022). Sensing Nitriles with THz Spectroscopy of Urine Vapours from Cancers Patients Subject to Chemotherapy. Sci. Rep..

[46] Vaks V., Chemyaeva M., Anfertev V., Domracheva E., Garanina O., Pripolzin S., Yablokov A. (2019). The Application of High Resolution Terahertz Gas Spectroscopy for Medical Diagnostics Based on the Analysis of Exhaled Breath and Biological Liquid Vapor. ITM Web of Conferences.

[47] Fosnight A.M., Moran B.L., Medvedev I.R. (2013). Chemical Analysis of Exhaled Human Breath Using a Terahertz Spectroscopic Approach. Appl. Phys. Lett..

[48] Hindle F., Kuuliala L., Mouelhi M., Cuisset A., Bray C., Vanwolleghem M., Devlieghere F., Mouret G., Bocquet R. (2018). Monitoring of Food Spoilage by High Resolution THz Analysis. Analyst.

[49] Hindle F., Kuuliala L., Mouelhi M., Cuisset A., Bray C., Vanwolleghem M., Devlieghere F., Mouret G., Bocquet R. (2019). Spoilage of Salmon Fillets as Observed by THz Waves. Proceedings of the 2019 44th International Conference on Infrared, Millimeter, and Terahertz Waves (IRMMW-THz).

[50] Bassi J., Stringer M., Miles B., Zhang Y. (2009). Terahertz Time-Domain Spectroscopy of High-Pressure Flames. Front. Energy Power Eng. China.

[51] Zhan H., Li Q., Zhao K., Zhang L., Zhang Z., Zhang C., Xiao L. (2015). Evaluating PM2.5 at a Construction Site Using Terahertz Radiation. IEEE Trans. Terahertz Sci. Technol..

[52] Smith R.M., Arnold M.A. (2015). Selectivity of Terahertz Gas-Phase Spectroscopy. Anal. Chem..

[53] Lin H., Withayachumnankul W., Fischer B.M., Mickan S.P., Abbott D. Gas Recognition with Terahertz Time-Domain Spectroscopy and Reference-Free Spectrum: A Preliminary Study. Proceedings of the 2008 33rd International Conference on Infrared, Millimeter and Terahertz Waves.

[54] Tonouchi M. (2007). Cutting-Edge Terahertz Technology. Nat. Photonics.

[55] Araki M., Tabata Y., Shimizu N., Matsuyama K. (2019). Terahertz Spectroscopy of CO and NO: The First Step toward Temperature and Concentration Detection for Combustion Gases in Fire Environments. J. Mol. Spectrosc..

[56] Zhang D., Qu J., Ouyang Y., Li S., Song Y. (2022). Transmission Characteristics of Terahertz Imaging Detection in Smoke Environments. Fire Technol..

[57] Chattopadhyay G., Reck T., Tang A., Jung-Kubiak C., Lee C., Siles J., Schlecht E., Kim Y.M., Chang M.-C.F., Mehdi I. (2015). Compact Terahertz Instruments for Planetary Missions.

[58] Hindle F., Cuisset A., Bocquet R., Mouret G. (2008). Continuous-Wave Terahertz by Photomixing: Applications to Gas Phase Pollutant Detection and Quantification. Comptes Rendus Phys..

[59] Puc U., Bach T., Günter P., Zgonik M., Jazbinsek M. (2021). Ultra-Broadband and High-Dynamic-Range THz Time-Domain Spectroscopy System Based on Organic Crystal Emitter and Detector in Transmission and Reflection Geometry. Adv. Photonics Res..

[60] Puc U., Bach T., Michel V., Zgonik M., Medrano C., Jazbinsek M. DSTMS-Based Ultrabroadband Terahertz Time-Domain Spectroscopy. Proceedings of the 2019 Conference on Lasers and Electro-Optics Europe & European Quantum Electronics Conference (CLEO/Europe-EQEC).

[61] Uno T., Tabata H. (2010). In Situ Measurement of Combustion Gas Using Terahertz Time Domain Spectroscopy Setup for Gas Phase Spectroscopy and Measurement of Solid Sample. Jpn. J. Appl. Phys..

[62] Yang Y., Mandehgar M., Grischkowsky D.R. (2012). Understanding THz Pulse Propagation in the Atmosphere. IEEE Trans. Terahertz Sci. Technol..

[63] Zhang X.-C., Xu J. (2010). Generation and Detection of THz Waves. Introduction to THz Wave Photonics.

[64] Dhillon S.S., Vitiello M.S., Linfield E.H., Davies A.G., Hoffmann M.C., Booske J., Paoloni C., Gensch M., Weightman P., Williams G.P. (2017). The 2017 Terahertz Science and Technology Roadmap. J. Phys. Appl. Phys..

[65] Jepsen P.U., Cooke D.G., Koch M. (2010). Terahertz Spectroscopy and Imaging—Modern Techniques and Applications. Laser Photonics Rev..

[66] Tomasino A., Parisi A., Stivala S., Livreri P., Cino A.C., Busacca A.C., Peccianti M., Morandotti R. (2013). Wideband THz Time Domain Spectroscopy Based on Optical Rectification and Electro-Optic Sampling. Sci. Rep..

[67] Lu X., Zhang X.-C. (2014). Investigation of Ultra-Broadband Terahertz Time-Domain Spectroscopy with Terahertz Wave Gas Photonics. Front. Optoelectron..

[68] Somma C., Folpini G., Gupta J., Reimann K., Woerner M., Elsaesser T. (2015). Ultra-Broadband Terahertz Pulses Generated in the Organic Crystal DSTMS. Opt. Lett..

[69] Lee S.-H., Jazbinsek M., Hauri C.P., Kwon O.-P. (2016). Recent Progress in Acentric Core Structures for Highly Efficient Nonlinear Optical Crystals and Their Supramolecular Interactions and Terahertz Applications. CrystEngComm.

[70] Hagelschuer T., Wienold M., Richter H., Schrottke L., Grahn H.T., Hübers H.-W. (2017). Real-Time Gas Sensing Based on Optical Feedback in a Terahertz Quantum-Cascade Laser. Opt. Express.

[71] Lee Y.-S. (2009). Continuous-Wave Terahertz Sources and Detectors. Principles of Terahertz Science and Technology.

[72] Tan P., Huang J., Liu K., Xiong Y., Fan M. (2012). Terahertz Radiation Sources Based on Free Electron Lasers and Their Applications. Sci. China Inf. Sci..

[73] Xiang Y., Zhu J., Wu L., You Q., Ruan B., Dai X. (2018). Highly Sensitive Terahertz Gas Sensor Based on Surface Plasmon Resonance With Graphene. IEEE Photonics J..

[74] Chen T., Han Z., Liu J., Hong Z. (2014). Terahertz Gas Sensing Based on a Simple One-Dimensional Photonic Crystal Cavity with High-Quality Factors. Appl. Opt..

[75] Hindle F., Bocquet R., Pienkina A., Cuisset A., Mouret G. (2019). Terahertz Gas Phase Spectroscopy Using a High-Finesse Fabry–Pérot Cavity. Optica.

[76] Elmaleh C., Simon F., Decker J., Dumont J., Cazier F., Fourmentin M., Bocquet R., Cuisset A., Mouret G., Hindle F. (2023). THz Cavity Ring-down Quantitative Gas Phase Spectroscopy. Talanta.

[77] Kumar P., Sharma A.K., Prajapati Y.K. (2022). Graphene-Based Plasmonic Sensor at THz Frequency with Photonic Spin Hall Effect Assisted by Magneto-Optic Phenomenon. Plasmonics.

[78] You B., Lu J.-Y., Bahadar Khan S., Asiri A.M., Akhtar K. (2020). Optical Gas Sensors Using Terahertz Waves in the Layered Media. Gas Sensors.

[79] Serita K., Murakami H., Kawayama I., Tonouchi M. (2019). A Terahertz-Microfluidic Chip with a Few Arrays of Asymmetric Meta-Atoms for the Ultra-Trace Sensing of Solutions. Photonics.

[80] Li Z., Rothbart N., Deng X., Geng H., Zheng X., Neumaier P., Hübers H.-W. (2020). Qualitative and Quantitative Analysis of Terahertz Gas-Phase Spectroscopy Using Independent Component Analysis. Chemom. Intell. Lab. Syst..

[81] Farhad A., Pyun J.-Y. (2023). Terahertz Meets AI: The State of the Art. Sensors.

[82] Wang W., Zhu N., Wang Z., Zhao C., Song Z., Chen X., Chao X. (2022). Efficient Terahertz Absorption Gas Sensor with Gaussian Process Regression in Time- and Frequency-Domain. Sens. Actuators B Chem..

[83] Mittleman D.M., Jacobsen R.H., Neelamani R., Baraniuk R.G., Nuss M.C. (1998). Gas Sensing Using Terahertz Time-Domain Spectroscopy. Appl. Phys. B-Lasers Opt..

[84] Neese C.F., Medvedev I.R., Plummer G.M., Frank A.J., Ball C.D., Lucia F.C.D. (2012). Compact Submillimeter/Terahertz Gas Sensor With Efficient Gas Collection, Preconcentration, and Ppt Sensitivity. IEEE Sens. J..

[85] Zhang W., Nickel D., Mittleman D. (2017). High-Pressure Cell for Terahertz Time-Domain Spectroscopy. Opt. Express.

[86] Neumaier P., Schmalz K., Borngräber J., Kissinger D., Hübers H.W. Terahertz Gas-Sensors: Gas-Phase Spectroscopy and Multivariate Analysis for Medical and Security Applications. Proceedings of the 2015 IEEE SENSORS.

[87] Kilcullen P., Hartley I.D., Jensen E.T., Reid M. (2015). Terahertz Time Domain Gas-Phase Spectroscopy of Carbon Monoxide. J. Infrared Millim. Terahertz Waves.

[88] Sang B.H., Jeon T.-I. (2016). Pressure-Dependent Refractive Indices of Gases by THz Time-Domain Spectroscopy. Opt. Express.

[89] Foltynowicz R.J., Wanke M.C., Mangan M.A. (2005). Atmospheric Propagation of THz Radiation.

[90] Vaks V., Domracheva E., Chernyaeva M. THz Analyzers for Breath Research. Proceedings of the 2016 21st International Conference on Microwave, Radar and Wireless Communications (MIKON).

[91] Zhang J., Grischkowsky D. (2004). Terahertz Time-Domain Spectroscopy Study of Silica Aerogels and Adsorbed Molecular Vapors. J. Phys. Chem. B.

[92] Theuer M., Harsha S.S., Molter D., Torosyan G., Beigang R. (2011). Terahertz Time-Domain Spectroscopy of Gases, Liquids, and Solids. Chemphyschem.

[93] Waters J. (1992). Submillimeter-Wavelength Heterodyne Spectroscopy and Remote-Sensing of the Upper-Atmosphere. Proc. IEEE.

[94] Voitsekhovskaya O.K., Egorov O.V. (2013). The Absorption of Sulfur Dioxide in the Terahertz Range at Temperatures of 300–1200 K. Mosc. Univ. Phys. Bull..

[95] Yao J., Wang R., Cui H., Wang J., Escalante B. (2012). Atmospheric Propagation of Terahertz Radiation. Remote Sensing—Advanced Techniques and Platforms.

[96] Yang Y., Shutler A., Grischkowsky D. (2011). Measurement of the Transmission of the Atmosphere from 0.2 to 2 THz. Opt. Express.

[97] Slocum D.M., Slingerland E.J., Giles R.H., Goyette T.M. (2013). Atmospheric Absorption of Terahertz Radiation and Water Vapor Continuum Effects. J. Quant. Spectrosc. Radiat. Transf..

[98] Yang Y., Mandehgar M., Grischkowsky D.R. (2011). Broadband THz Pulse Transmission through the Atmosphere. IEEE Trans. Terahertz Sci. Technol..

[99] Vogt D.W., Jones A.H., Leonhardt R. (2020). Terahertz Gas-Phase Spectroscopy Using a Sub-Wavelength Thick Ultrahigh-Q Microresonator. Sensors.

[100] Xin X., Altan H., Saint A., Matten D., Alfano R.R. (2006). Terahertz Absorption Spectrum of Para and Ortho Water Vapors at Different Humidities at Room Temperature. J. Appl. Phys..

[101] Sun H., Yang Z., Kinev N.V., Kiselev O.S., Lv Y., Huang Y., Hao L., Zhou X., Ji M., Tu X. (2017). Terahertz Spectroscopy of Dilute Gases Using Bi_2_Sr_2_CaCu_2_O_8+δ_ Intrinsic Josephson-Junction Stacks. Phys. Rev. Appl..

[102] Bigourd D., Mouret G., Cuisset A., Hindle F., Fertein E., Bocquet R. (2008). Rotational Spectroscopy and Dynamics of Carbonyl Sulphide Studied by Terahertz Free Induction Decays Signals. Opt. Commun..

[103] Melinger J.S., Harsha S.S., Laman N., Grischkowsky D. (2010). Temperature Dependent Characterization of Terahertz Vibrations of Explosives and Related Threat Materials. Opt. Express.

[104] Cai H., Wang D., Shen J. (2013). Study on Terahertz Spectra of SO_2_ and H_2_S. Sci. China Phys. Mech. Astron..

[105] Pacheco-Torgal F., Jalali S. (2011). Nanotechnology: Advantages and Drawbacks in the Field of Construction and Building Materials. Constr. Build. Mater..

[106] Varol Y., Öner C., Öztop H.F., Altun Ş. (2014). Comparison of Methanol, Ethanol, or n-Butanol Blending with Unleaded Gasoline on Exhaust Emissions of an SI Engine. Energy Sources Part Recovery Util. Environ. Eff..

[107] Zhu J., Zhan H.L., Miao X.Y., Song Y., Zhao K. (2016). Adsorption Dynamics and Rate Assessment of Volatile Organic Compounds in Active Carbon. Phys. Chem. Chem. Phys. PCCP.

[108] Graber B., Kim C., Wu D.H. (2017). High SNR Single Measurements of Trace Gas Phase Spectra at THz Frequencies. Appl. Phys. Lett..

[109] Lin S., Liu W., Hou X., Peng Z., Chen Z., Hu F. (2023). Specific Detection of N-Propanol Gas via Terahertz Metasurface Sensor Modified by Molecularly Imprinted Polymer. Spectrochim. Acta. A Mol. Biomol. Spectrosc..

[110] Altemose B., Gong J., Zhu T., Hu M., Zhang L., Cheng H., Zhang L., Tong J., Kipen H.M., Strickland P.O. (2015). Aldehydes in Relation to Air Pollution Sources: A Case Study around the Beijing Olympics. Atmos. Environ..

[111] Harde H., Zhao J., Wolff M., Cheville R.A., Grischkowsky D. (2001). THz Time-Domain Spectroscopy on Ammonia. J. Phys. Chem. A.

[112] Tang N., Hattori T., Taga R., Igarashi K., Yang X., Tamura K., Kakimoto H., Mishukov V.F., Toriba A., Kizu R. (2005). Polycyclic Aromatic Hydrocarbons and Nitropolycyclic Aromatic Hydrocarbons in Urban Air Particulates and Their Relationship to Emission Sources in the Pan–Japan Sea Countries. Atmos. Environ..

[113] Gupta S., Pathak B., Fulekar M.H. (2015). Molecular Approaches for Biodegradation of Polycyclic Aromatic Hydrocarbon Compounds: A Review. Rev. Environ. Sci. Biotechnol..

[114] Du Y., Fang H., Zhang Q., Hong Z. Terahertz Spectroscopic Characterization of Naphthalene and 1-Nitronaphthalene. Proceedings of the 2015 40th International Conference on Infrared, Millimeter, and Terahertz waves (IRMMW-THz).

[115] Cataldo F., Angelini G., Aníbal García-Hernández D., Manchado A. (2013). Far Infrared (Terahertz) Spectroscopy of a Series of Polycyclic Aromatic Hydrocarbons and Application to Structure Interpretation of Asphaltenes and Related Compounds. Spectrochim. Acta. A Mol. Biomol. Spectrosc..

[116] Han J., Xu H., Zhu Z., Yu X., Li W. (2004). Terahertz Spectroscopy of Naphthalene, α-Naphthol, β-Naphthol, Biphenyl and Anthracene. Chem. Phys. Lett..

[117] Luengas A., Barona A., Hort C., Gallastegui G., Platel V., Elias A. (2015). A Review of Indoor Air Treatment Technologies. Rev. Environ. Sci. Biotechnol..

[118] Allodi M.A., Ioppolo S., Kelley M.J., McGuire B.A., Blake G.A. (2014). The Structure and Dynamics of Carbon Dioxide and Water Containing Ices Investigated via THz and Mid-IR Spectroscopy. Phys. Chem. Chem. Phys..

[119] Shimizu N., Matsuyama K., Hosako I. Absorption Spectra of Hydrogen Chloride and Carbon Monoxide in Smoke. Proceedings of the 2012 37th International Conference on Infrared, Millimeter, and Terahertz Waves.

[120] Su Y., Zheng X., Deng X. (2017). Terahertz Spectrum Analysis Based on Empirical Mode Decomposition. J. Infrared Millim. Terahertz Waves.

[121] Nolt I.G., Radostitz J.V., DiLonardo G., Evenson K.M., Jennings D.A., Leopold K.R., Vanek M.D., Zink L.R., Hinz A., Chance K.V. (1987). Accurate Rotational Constants of CO, HCl, and HF: Spectral Standards for the 0.3- to 6-THz (10- to 200-cm^−1^) Region. J. Mol. Spectrosc..

[122] Harmon S.A., Cheville R.A. (2004). Part-per-Million Gas Detection from Long-Baseline THz Spectroscopy. Appl. Phys. Lett..

[123] Harde H., Katzenellenbogen N., Grischkowsky D. (1994). Terahertz Coherent Transients from Methyl Chloride Vapor. JOSA B.

[124] Bereiter B., Kawamura K., Severinghaus J.P. (2018). New Methods for Measuring Atmospheric Heavy Noble Gas Isotope and Elemental Ratios in Ice Core Samples. Rapid Commun. Mass Spectrom. RCM.

[125] Jaszczak E., Polkowska Ż., Narkowicz S., Namieśnik J. (2017). Cyanides in the Environment—Analysis—Problems and Challenges. Environ. Sci. Pollut. Res. Int..

[126] Shimizu N., Kikuchi K., Ikari T., Matsuyama K., Wakatsuki A., Kohjiro S., Fukasawa R. (2011). Absorption Spectra of Smoke Emitted from Heated Nylon Fabric Measured with a Continuous-Wave Sub-Terahertz Spectrometer. Appl. Phys. Express.

[127] Sitnikov D.S., Romashevskiy S.A., Pronkin A.A., Ilina I.V. (2019). Open-Path Gas Detection Using Terahertz Time-Domain Spectroscopy. J. Phys. Conf. Ser..

[128] Badr O., Probert S.D. (1993). Environmental Impacts of Atmospheric Nitrous Oxide. Appl. Energy.

[129] Hill M.K. (2010). Understanding Environmental Pollution.

[130] Drouin B.J., Maiwald F.W. (2006). Extended THz Measurements of Nitrous Oxide, N_2_O. J. Mol. Spectrosc..

[131] Kim G.-R., Lee H.-B., Jeon T.-I. (2020). Terahertz Time-Domain Spectroscopy of Low-Concentration N_2_O Using Long-Range Multipass Gas Cell. IEEE Trans. Terahertz Sci. Technol..

[132] Morino I., Yamada K.M.T., Maki A.G. (1999). Terahertz Measurements of Rotational Transitions in Vibrationally Excited States of N_2_O. J. Mol. Spectrosc..

[133] Müller H.S.P., Kobayashi K., Takahashi K., Tomaru K., Matsushima F. (2015). Terahertz Spectroscopy of N_18_O and Isotopic Invariant Fit of Several Nitric Oxide Isotopologs. J. Mol. Spectrosc..

[134] Drouin B.J., Crawford T.J., Yu S. (2017). Validation of Ozone Intensities at 10 µm with THz Spectrometry. J. Quant. Spectrosc. Radiat. Transf..

[135] Xu J., Schreier F., Loyola D., Schüssler O., Doicu A., Trautmann T. Monitoring Ozone in Different Spectral Regimes from Space and Balloon (Sentinel-4/-5P, TELIS). Proceedings of the 2016 IEEE International Geoscience and Remote Sensing Symposium (IGARSS).

[136] Li Q., Zhao K., Zhang L., Liang C., Zhang Z., Zhang C., Han D. (2014). Probing PM2.5 with Terahertz Wave. Sci. China Phys. Mech. Astron..

[137] Li N., Zhan H., Zhao K., Zhang Z., Li C., Zhang C. (2016). Characterizing PM2.5 in Beijing and Shanxi Province Using Terahertz Radiation. Front. Optoelectron..

[138] Frank N.H. (2006). Retained Nitrate, Hydrated Sulfates, and Carbonaceous Mass in Federal Reference Method Fine Particulate Matter for Six Eastern U.S. Cities. J. Air Waste Manag. Assoc..

[139] Rabbani K.A., Charles W., Cord-Ruwisch R., Ho G. (2015). Recovery of Sulphur from Contaminated Air in Wastewater Treatment Plants by Biofiltration: A Critical Review. Rev. Environ. Sci. Biotechnol..

[140] Krayzelova L., Bartacek J., Díaz I., Jeison D., Volcke E.I.P., Jenicek P. (2015). Microaeration for Hydrogen Sulfide Removal during Anaerobic Treatment: A Review. Rev. Environ. Sci. Biotechnol..

[141] Matton S., Rohart F., Bocquet R., Mouret G., Bigourd D., Cuisset A., Hindle F. (2006). Terahertz Spectroscopy Applied to the Measurement of Strengths and Self-Broadening Coefficients for High-J Lines of OCS. J. Mol. Spectrosc..

[142] Cazzoli G., Cludi L., Cotti G., Esposti C.D., Dore L. (1994). Far Infrared Spectrum of SO in the 3Σ and 1Δ Electronic States. J. Mol. Spectrosc..

[143] Martin-Drumel M.A., Pirali O., Eliet S., Cuisset A. (2012). High Resolution Far Infrared Laboratory Spectroscopy of Transient Species: Application to the SO Radical (X3Σ). EAS Publ. Ser..

[144] Martin-Drumel M.A., Hindle F., Mouret G., Cuisset A., Cernicharo J. (2015). A Complete Spectroscopic Characterization of SO and Its Isotopologues up to the Terahertz Domain. Astrophys. J..

[145] von Kienle H., Kunze N., Mertens D.H. (1994). The Use of Activated Carbon in the Removal of VOC’s. Stud. Environ. Sci..

[146] Mohamed F., Kim J., Huang R., Nu H.T., Lorenzo V. (2014). Efficient Control of Odors and VOC Emissions via Activated Carbon Technology. Water Environ. Res..

[147] Zhu J., Zhan H., Miao X., Zhao K., Zhou Q. (2017). Terahertz Double-Exponential Model for Adsorption of Volatile Organic Compounds in Active Carbon. J. Phys. Appl. Phys..

[148] You B., Ho C.-H., Zheng W.-J., Lu J.-Y. (2015). Terahertz Volatile Gas Sensing by Using Polymer Microporous Membranes. Opt. Express.

[149] Phillips M., Herrera J., Krishnan S., Zain M., Greenberg J., Cataneo R.N. (1999). Variation in Volatile Organic Compounds in the Breath of Normal Humans. J. Chromatogr. B Biomed. Sci. Appl..

[150] Komatsu K., Iwamoto T., Ito H., Saitoh H. (2022). THz Gas Sensing Using Terahertz Time-Domain Spectroscopy with Ceramic Architecture. ACS Omega.

[151] Vaks V., Anfertev V., Chernyaeva M., Domracheva E., Shcherbatyuk T.G., Zhukova E.S. (2022). Analysis of Rodent’s Urine Vapors with Using THz High Resolution Spectrometer for Revealing the Markers of Dysbacteriosis. Int. Conf. Adv. Laser Technol. ALT.

[152] Vaks V., Aizenshtadt A., Anfertev V., Chernyaeva M., Domracheva E., Gavrilova K., Larin R., Pripolzin S., Shakhova M. (2021). Analysis of the Thermal Decomposition Products of Pathological and Healthy Tissues in Paranasal Sinuses: A High-Resolution Terahertz Gas Spectroscopy Study. Appl. Sci..

[153] Vaks V.L., Domracheva E.G., Chernyaeva M.B., Anfertev V.A., Maslennikova A.V., Zheleznyak A.V., Knyazeva T.D., Rodionov M.A., Maiorov A.I. (2022). Application of a High-Resolution Terahertz Gas Spectroscopy Method to Compositional Analysis of Thermal Decomposition Products of Human Fluids (Urine). J. Opt. Technol..

[154] Yasui T., Kawamoto K., Hsieh Y.-D., Sakaguchi Y., Jewariya M., Inaba H., Minoshima K., Hindle F., Araki T. (2012). Enhancement of Spectral Resolution and Accuracy in Asynchronous-Optical-Sampling Terahertz Time-Domain Spectroscopy for Low-Pressure Gas-Phase Analysis. Opt. Express.

[155] Kim G.-J., Jeon S.-G., Kim J.-I., Jin Y.-S. (2008). High Speed Scanning of Terahertz Pulse by a Rotary Optical Delay Line. Rev. Sci. Instrum..

[156] Burford N.M., El-Shenawee M.O. (2017). Review of Terahertz Photoconductive Antenna Technology. Opt. Eng..

[157] Passarelli A., Rice T.E., Chowdhury M.A.Z., Powers M.N., Mansha M.W., Wilke I., Hella M.M., Oehlschlaeger M.A. (2022). Terahertz-Wave Absorption Gas Sensing for Dimethyl Sulfoxide. Appl. Sci..

